# Gaps between fruit and vegetable production, demand, and recommended consumption at global and national levels: an integrated modelling study

**DOI:** 10.1016/S2542-5196(19)30095-6

**Published:** 2019-07

**Authors:** Daniel Mason-D'Croz, Jessica R Bogard, Timothy B Sulser, Nicola Cenacchi, Shahnila Dunston, Mario Herrero, Keith Wiebe

**Affiliations:** a Commonwealth Scientific and Industrial Research Organisation, Agriculture and Food, St Lucia, QLD, Australia; b International Food Policy Research Institute, Environment and Production Technology, Washington, DC, USA

## Abstract

**Background:**

Current diets are detrimental to both human and planetary health and shifting towards more balanced, predominantly plant-based diets is seen as crucial to improving both. Low fruit and vegetable consumption is itself a major nutritional problem. We aim to better quantify the gap between future fruit and vegetable supply and recommended consumption levels by exploring the interactions between supply and demand in more than 150 countries from 1961 to 2050.

**Methods:**

In this global analysis, we use the International Model for Policy Analysis of Agricultural Commodities and Trade, which simulates the global agricultural sector, to explore the role of insufficient production of fruits and vegetables and the effects of food waste and public policy in achieving recommended fruit and vegetable consumption. First, we estimate the average historical (1961–2010) and future (2010–50) national consumption levels needed to meet WHO targets (a minimum target of 400 g/person per day or age-specific recommendations of 330–600 g/person per day) using population pyramids; for future consumption, we use projections from the Shared Socioeconomic Pathways (SSPs), a set of global socioeconomic scenarios characterised by varied assumptions on economic and population growth. We then simulate future fruit and vegetable production and demand to 2050 under three such scenarios (SSP1–3) to assess the potential impacts of economic, demographic, and technological change on consumer and producer behaviour. We then explore the potential effects of food waste applying various waste assumptions (0–33% waste). Finally, we apply two policy analysis frameworks (the NOURISHING framework and the Nuffield ladder) to assess the current state of public policy designed to achieve healthy diets.

**Findings:**

Historically, fruit and vegetable availability has consistently been insufficient to supply recommended consumption levels. By 2015, 81 countries representing 55% of the global population had average fruit and vegetable availability above WHO's minimum target. Under more stringent age-specific recommendations, only 40 countries representing 36% of the global population had adequate availability. Although economic growth will help to increase fruit and vegetable availability in the future, particularly in lower-income countries, this alone will be insufficient. Even under the most optimistic socioeconomic scenarios (excluding food waste), many countries fail to achieve sufficient fruit and vegetable availability to meet even the minimum recommended target. Sub-Saharan Africa is a particular region of concern, with projections suggesting, by 2050, between 0·8 and 1·9 billion people could live in countries with average fruit and vegetable availability below 400 g/person per day. Food waste is a serious obstacle that could erode projected gains. Assuming 33% waste and socioeconomic trends similar to historical patterns, the global average availability in 2050 falls below age-specific recommendations, increasing the number of people living in countries with insufficient supply of fruits and vegetables by 1·5 billion compared with a zero waste scenario.

**Interpretation:**

Increasing fruit and vegetable consumption is an important component of a shift towards healthier and more sustainable diets. Economic modelling suggests that even under optimistic socioeconomic scenarios future supply will be insufficient to achieve recommended levels in many countries. Consequently, systematic public policy targeting the constraints to producing and consuming fruits and vegetables will be needed. This will require a portfolio of interventions and investments that focus on increasing fruit and vegetable production, developing technologies and practices to reduce waste without increasing the consumer cost, and increasing existing efforts to educate consumers on healthy diets.

**Funding:**

The Commonwealth Scientific and Industrial Research Organisation; Climate Change, Agriculture and Food Security (CGIAR) Research Program on Climate Change, Agriculture and Food Security; CGIAR Research Program on Policy, Institutions, and Markets; Bill & Melinda Gates Foundation; and Johns Hopkins University.

## Introduction

Continued progress across the food system, particularly since the green revolution, has boosted agricultural production to the point where calorie supply globally is outpacing recommended consumption levels. This has led to a decades-long decline in hunger rates, despite recent upticks caused primarily by state fragility.^1^ This reduction in hunger, however, has been achieved with substantial impacts on the environment, threatening the sustainability of our gains.^2^ The gains have also come at a human health cost as diets globally have become more homogeneous^3^ and are increasingly characterised by cheap calories, highly processed foods, and overconsumption.^4^ The population with overweight and obesity now outnumber those with insufficient caloric intake, even as more than 2 billion people experience micronutrient deficiencies.^5^ Unbalanced diets are the reason many people in most countries have some form of malnutrition, due to the inadequate, unbalanced, or excessive consumption of macronutrients and micronutrients.^6^ This has led to the proliferation of many of the non-communicable diseases that are now the main cause of premature mortality globally.^7^

**Research in context**

**Evidence before this study**

Three recent high-level studies (the EAT–*Lancet* Commission on healthy diets from sustainable food systems, the *Lancet* Commission report on the Global Syndemic of Obesity, Undernutrition, and Climate Change, and the Global Nutrition Report) have summarised the literature on healthy and sustainable diets. All three studies highlighted the negative effects of current diets on human and planetary health and identified the need for radical change in the food system to improve diets to bridge the gap between current and recommended diets. Work done by WHO and in Global Burden of Disease assessments has consistently shown that low consumption of fruits and vegetables is a major obstacle to achieving healthy diets. Further studies have tried to assess the gap between future production of fruits and vegetables and recommended consumption levels. However, these studies projected supply in isolation from changing future food demand and did not directly simulate the inter-related nature of production, demand, and socioeconomic development. Several integrated modelling studies that have simulated future production and demand have found that economic growth alone would be insufficient to supply healthy diets or achieve food security but did not assess future gaps of fruit and vegetable production, demand, and recommended consumption levels.

**Added value of this study**

We expand on the evidence of low fruit and vegetable consumption and production by applying a global integrated economic model of the agriculture sector to simulate how fruit and vegetable demand and production could change under a range of alternative futures with different assumptions on socioeconomic and technological change. Applying state-of-the-art scenarios and economic modelling, we explore how consumers and producers in more than 150 countries could respond to changing market conditions in these scenarios to better project the gap between future fruit and vegetable production and recommended consumption levels at the global and national levels. We explore the uncertainty of food waste for fruits and vegetables with a range of waste assumptions. We show that even under more optimistic consumer waste scenarios, many countries will probably fail to supply sufficient fruits and vegetables to meet recommended consumption levels. Finally, we review current policies using both the NOURISHING framework and the Nuffield ladder to show the gap between public actions and what would be needed to achieve healthy fruit and vegetable consumption levels.

**Implications of all the available evidence**

Diets—and low fruit and vegetable consumption—are associated with the most important causes of premature mortality globally. Shifting towards healthier diets rich in nutritious foods such as fruits and vegetables could lead to a win-win scenario for public and ecological health. Historical trends suggest that economic growth might help to increase fruit and vegetable consumption, but without substantial public efforts, this increase is likely to fall short of meeting the required increases. Achieving recommended consumption levels will require concentrated efforts across the food system to reorient investments and interventions to prioritise fruits and vegetables more. It will require additional investments in research and development to encourage more fruit and vegetable production, while decreasing its environmental footprint, as well as new processing, storage, and distribution technologies to reduce waste. Targeted fiscal policies such as price supports and procurement policies should also be considered to supplement public awareness efforts to incentivise consumer behaviour change.

Increasingly, it is recognised that the global food system must move its focus from quantity towards dietary quality and health and environmental outcomes.^5^, ^8^ To this end, much attention has been focused on reducing negative dietary risk factors such as overconsumption of processed or discretionary foods, sugar, saturated fat, and red meat.^2^, ^9^ These studies also suggest public health and the environment would benefit from rebalancing towards more plant-based diets, with increased fruit and vegetable consumption crucial.^5^ Several studies have also suggested that current and projected fruit and vegetable production will fail to meet healthy consumption levels^10^, ^11^ but have not directly simulated the interconnected nature of food production, demand, socioeconomic development, and food prices—a major determinant of consumer behaviour.

This study aims to build on these previous studies by quantifying the gap between future fruit and vegetable supply, demand, and recommended consumption levels. First, we assess the historical gap between supply and recommended consumption levels. We then explore future uncertainty of both supply and demand using a range of alternative socioeconomic scenarios. To better simulate the interconnected nature of technology development, population growth, economic development on both agricultural production and food demand, we simulate these scenarios in the International Model for Policy Analysis of Agricultural Commodities and Trade (IMPACT), a global economic model of the agriculture sector. We then consider the potential challenges consumer waste might present in the future. Finally, we review public policy targeting fruits and vegetables and analyse them using multiple policy frameworks to determine the scope and scale of current policies in this space.

## Methods

### Scenario analysis and selection

Projection of future fruit and vegetable availability involves substantial uncertainty, as the global food system is highly interconnected and interdependent. Future fruit and vegetable availability will be affected by many factors including economic development, population growth, changing consumer behaviour, and climate change, to name a few. In this study, we focus on assessing the impact of varying levels of socioeconomic development, while recognising that these other factors are important.

Here, we use exploratory scenarios that present plausible future pathways, based on internally consistent assumptions of various drivers, trends, and interactions into the future.^12^ We chose this scenario analysis approach due to the fundamental uncertainty in projecting complex systems with interconnected and dependent variables; in such cases, the future possibility space is too large to be able to reasonably estimate confidence intervals. Stochastic analysis, which can be done in a forecasting exercise, attempts to predict the future on the basis of past behaviour and relationships. However, given that the future will potentially be very different than the past, this type of exercise will almost certainly consider a limited possibility space. Therefore, we explore future uncertainty using a range of scenarios that provide a broader envelope of plausible futures than would be possible solely by extrapolating from past behaviour. Specifically, we use the Shared Socioeconomic Pathways (SSPs), a set of global socioeconomic scenarios developed for the International Panel on Climate Change.^13^ We use three of the SSPs (1–3) that provide a broad envelope of plausible pathways to 2050, characterised by varied assumptions on economic and population growth. Of the three SSPs considered, SSP 1 is the most optimistic scenario envisioning a more sustainable development pathway with a global population by 2050 of 8·5 billion people with per-capita gross domestic product (GDP) of US$34 000; SSP 2 is a middle-of-the-road scenario, where global population grows to 9·2 billion people with per-capita GDP of $25 000; and SSP 3 is the most pessimistic scenario with global population reaching nearly 10 billion people with a per-capita GDP of $18 000 (see appendix pp 18–19). We assume IMPACT's default dietary pathways,^14^ which are based on historical relationships between food demand and income growth, to allow us to estimate the future availability gap assuming business-as-usual policies and dietary transitions.

### Economic modelling

To simulate the complex connections across the global agricultural system, we applied the IMPACT model (version 3.3). IMPACT is an integrated modelling system composed of an ensemble of economic and biophysical models, which allows it to simulate agricultural production in the face of biophysical and economic constraints. It has been extensively used in a series of global agricultural assessments and modelling exercises that have considered the impacts of policies and production shocks on the global food system.^15^, ^16^, ^17^ In the class of global economic simulation models, IMPACT is the most disaggregated in terms of country and commodity representation. At its core, IMPACT is a global partial-equilibrium multimarket model that simulates national and global agricultural production, demand, and trade for 158 countries or regions and 62 commodities.^18^ Of the 62 commodities simulated in IMPACT, three commodities are fruits and one commodity is vegetables. For this analysis, we additionally grouped the 158 countries and regions into seven geographical regions (east Asia and Pacific, Europe and North America, the former Soviet Union [including the Baltic states up to 1990, after which they are considered to be part of Europe], Latin America and Caribbean, Middle East and north Africa, south Asia, and sub-Saharan Africa) and two income groups (developed and developing), where appropriate. Summaries of the geographical and commodity scope of IMPACT, including a mapping of the Food and Agriculture Organization (FAO) of the UN's fruit and vegetable commodities to IMPACT commodities, are presented in the appendix (p 10).

IMPACT simulates the interrelated nature of markets, which can respond to changes in production technologies, climate, consumer preferences, demographics, and public policy. Household demand is simulated through the interaction of a mix of exogenous (population, economic growth) and endogenous (prices) factors with consumer preferences represented by income and price elasticities following historical relationships (eg, Engel's and Bennett's laws^17^). Similarly, producer behaviour is simulated with a mix of exogenous and endogenous factors, where producers respond endogenously to changing price levels, as well as to exogenous assumptions on technological developments. Production and demand within countries are linked through trade and international commodity markets, with IMPACT finding an equilibrium such that commodity prices clear all commodity markets (ie, global supply equals global demand). The model, therefore, can consistently simulate many of the crucial interactions between consumers, producers, and distribution networks.

IMPACT does not simulate actual consumption but it does simulate average food availability, from which we can estimate calorie availability and average consumption through post-solution application of food waste assumptions, as has been done in several recent health and nutrition assessments.^9^, ^16^, ^19^, ^20^ A more detailed description of IMPACT is available in the appendix, with the complete model documentation available online.^18^

### Defining recommended consumption levels

We use two WHO targets to provide the recommended fruit and vegetable consumption levels in this analysis. The first is a minimum recommendation of 400 g/person per day,^21^ similar to studies that suggest five servings of fruits and vegetables can provide the bulk of health benefits.^22^ The second is an age-adjusted recommendation, which is based on health modelling used to estimate a minimum dietary risk distribution—ie, to reduce the global risk of ischaemic heart disease, cerebrovascular disease, lung cancer, and selected gastrointestinal cancers. This more rigorous recommendation suggests 600 g per day for adults and adolescents (aged ≥15 years), 480 g per day for children aged 5–14 years, and 330 g per day for children aged 0–4 years.^23^ Both WHO recommendations are broadly consistent with national recommendations based on detailed health modelling (appendix p 1).^24^

We estimate average recommended consumption levels using population statistics or projections to estimate the average per-capita consumption level. For historical age-adjusted recommendations, we use age-specific population pyramids from the UN Population Prospects^25^ and for future projections, we use population projections from the SSPs.^26^ In both cases, we use age-specific numbers to calculate a population-weighted average consumption level by country that would be consistent with the WHO recommended consumption levels and the respective population pyramid. This application of age-specific recommendations allows us to take into account projected regional demographic changes.

### Estimating fruit and vegetable availability

To estimate historical (1961–2010) fruit and vegetable availability, we use total food demand of fruits and vegetables from the FAO's commodity balance sheets.^27^ The FAO reports for 17 fruit and vegetable items, with many of these representing aggregations (eg, citrus, other). Data are reported in primary commodity equivalence (eg, wheat flour is converted into equivalent quantities of wheat grain).^28^ We then use historical population statistics^25^ to estimate average fruit and vegetable availability in per-capita terms. The definition of fruits and vegetables used in this analysis follows those used by WHO and excludes pulses (eg, lentils, beans, etc) and starchy roots and tubers (eg, potatoes, cassava, etc). Future (2010–50) fruit and vegetable availability are taken from the scenario results simulated in IMPACT. IMPACT uses data from the FAO's commodity balance sheets and similarly represents agricultural production and demand in primary commodity equivalence. Due to data limitations on production, prices, and trade, IMPACT further aggregates fruits and vegetables into four commodities. Nevertheless, given that food dietary guidelines do not highly disaggregate recommended consumption levels, the model is still useful in assessing changes in production and demand with these targets.

### Incorporating food waste into the analysis

We include food waste in our analysis, recognising that some level of waste is inevitable and a certain amount of overproduction is necessary to ensure sufficient production to achieve recommended consumption levels. Food loss and waste estimates for fruits and vegetables vary substantially in the literature,^29^, ^30^, ^31^ in part due to varying definitions of what constitutes food losses and waste.^32^, ^33^ In this analysis, we define food waste as household food waste and treat other inefficiencies in the food system as food losses, which are accounted for in IMPACT in two ways: 1) post-harvest losses, which include activities at the point of primary production and are represented in IMPACT through reduced yields than what might be realised biologically; and 2) processing and storage losses, which are components of other demand and are included in IMPACT to ensure a full accounting of all primary production throughout the food value chain.^34^ Consumer or household food waste makes up the difference between the food demand reported in IMPACT and average food consumption. A visual representation of the varying stages of how food losses and waste are represented in IMPACT is included in the appendix (p 16).

Projecting future food waste is difficult, given the opposing historical relationship between higher incomes and increased waste^29^, ^32^ and growing attention to the need to reduce food waste if society is to achieve a sustainable food system.^5^ To account for this uncertainty, we apply stylised waste scenarios to estimate the quantity of additional fruits and vegetables that could be needed to ensure adequate future supply. First, we use the FAO's most recent region-specific estimates from 2013,^31^ which have been used in several studies on global diets, health, and sustainability.^5^, ^9^, ^16^ These estimates range from 5% in sub-Saharan Africa to 28% in North America and Oceania (appendix p 16). To project potential future waste, we have also included two alternate waste assumptions of 15% and 33% that are projected across all regions, similar to Siegel and colleagues.^10^ This range is similar in scope to the regional differences found in the FAO estimates and allows us to consider futures where waste increases with further economic development (33% waste), as well as futures where efforts to reduce food waste are implemented (15% waste).

### Reviewing public policies concerning fruits and vegetables

Analysis of the current state of policies was based on the World Cancer Research Fund International's (WCRF) NOURISHING database.^35^ The WCRF maintains this global database of implemented government policy actions that is updated three to four times per year (when accessed, the most recent update was in October, 2018), using a two-stage search and review and verification process.^36^ The database is intended to be comprehensive in its scope, although is unlikely to reflect an exhaustive list of all implemented policies due to limitations in data availability. To the authors' knowledge, the database does, however, offer the best available global information source on implemented policies related to promotion of healthy diets and is the source of policy data used in other reputable global analyses such as the Global Nutrition Report.^37^ Policies related to fruits and vegetables were identified using keyword searches for “fruit” or “vegetable”. Only policy examples that specifically targeted fruits and vegetables were included. We defined policies targeting fruits and vegetables as those policies that either described a minimum quantity of fruits or vegetables to be provided or described an activity or intervention specifically promoting increased consumption of fruits or vegetables. This definition might exclude some policies that broadly target healthy eating but that did not specifically mention fruits or vegetables. In many cases, policies were identified at the regional level (eg, the EU) instead of at the country level. In these cases, we assumed that the policies were implemented by all countries within the regional group. The relevant regions and country members used in this analysis are the EU with 28 countries, the Caribbean Community group with 15 countries, and 22 countries in the Pacific Island Nations and Territories group.

We further complemented the NOURISHING framework^38^ by mapping the policy options to the Nuffield ladder, an alternative policy framework used to analyse the efficacy of policies towards reducing obesity.^39^ The Nuffield ladder categorises policies not on the basis of their sphere of influence but on how forceful the policies are in terms of intervening in the choice environment, ranging from “provide information” (lowest or least forceful rung) to “eliminate choice” (highest or most forceful rung). In mapping the policy options, we considered the likely effect of each option from the consumer perspective. For example, the “incentives for store-owners to locate in underserved areas” NOURISHING policy option was mapped to “enabling choice” rather than “guide choice with incentives” on the Nuffield ladder because the incentives are targeted at the store owner, whereas the outcome of the policy provides consumers with greater choices in accessing stores. A complete mapping of the policies in the NOURISHING database to the Nuffield ladder can be found in the appendix (pp 24–25).

### Role of the funding source

The funders of the study had no role in study design, data collection, data analysis, data interpretation, or writing of the report. The corresponding author had full access to all the data in the study and had final responsibility for the decision to submit for publication.

## Results

Statistics from the FAO show that the per-capita availability of fruits and vegetables has consistently been insufficient to supply recommended consumption levels.^27^ In 1965, only 29 countries, representing about 17% of the global population, achieved average per-capita fruit and vegetable availability consistent with the minimum target of 400 g/person per day (table). By 2015, this number had increased to 81 countries, representing just 55% of the global population. Under the more stringent, age-adjusted recommendations, although progress was observed over this time, only 40 countries, representing about 36% of the global population had sufficient availability by 2015. Complete country results for both the age specific and minimum WHO recommendations are available in the appendix (pp 23–24) and in an online repository.

**Table:** Regional summary of fruit and vegetable availability and progress towards availability of 400 g/person per day

	**1965**	**1990**	**2015**	**2050**		
				SSP1	SSP2	SSP3
**East Asia and Pacific**						
Fruit and vegetable availability (g/person per day)	194	318	834	982	924	877
Number of countries	22 (5)	22 (7)	23 (12)	23 (15)	23 (15)	23 (15)
Population (millions)	1862 (144)	3026 (271)	2242 (1886)	2173 (2105)	2261 (2186)	2352 (2266)
**South Asia**						
Fruit and vegetable availability (g/person per day)	167	198	326	1335	956	615
Number of countries	6 (0)	6 (0)	7 (0)	7 (4)	7 (3)	7 (2)
Population (millions)	637 (0)	1133 (0)	1747 (0)	2108 (2025)	2373 (1980)	2720 (2032)
Former Soviet Union*						
Fruit and vegetable availability (g/person per day)	292	349	526	678	644	624
Number of countries	1 (0)	1 (0)	12 (10)	12 (12)	12 (11)	12 (11)
Population (millions)	230 (0)	287 (0)	281 (270)	263 (263)	278 (270)	290 (279)
**Middle East and north Africa**						
Fruit and vegetable availability (g/person per day)	436	640	735	798	771	731
Number of countries	14 (7)	14 (11)	17 (15)	17 (15)	17 (15)	17 (15)
Population (millions)	145 (77)	291 (251)	499 (467)	647 (594)	716 (653)	809 (732)
**Sub-Saharan Africa**						
Fruit and vegetable availability (g/person per day)	178	178	206	355	301	248
Number of countries	38 (0)	38 (0)	43 (1)	43 (14)	43 (5)	43 (3)
Population (millions)	233 (0)	462 (0)	956 (10)	1542 (723)	1767 (519)	2055 (130)
**Latin America and Caribbean**						
Fruit and vegetable availability (g/person per day)	310	322	413	544	498	462
Number of countries	24 (6)	24 (5)	24 (13)	24 (18)	24 (16)	24 (14)
Population (millions)	250 (53)	441 (21)	616 (442)	675 (555)	742 (584)	854 (628)
Europe and North America*						
Fruit and vegetable availability (g/person per day)	454	601	632	700	689	708
Number of countries	27 (11)	27 (23)	31 (30)	31 (31)	31 (31)	31 (31)
Population (millions)	665 (393)	781 (757)	905 (898)	1053 (1053)	1027 (1027)	870 (870)
**Global**						
Fruit and vegetable availability (g/person per day)	252	335	546	862	732	608
Number of countries	132 (29)	132 (46)	158 (81)	158 (109)	158 (96)	158 (91)
Population (millions)	4019 (665)	6419 (1298)	7243 (3969)	8457 (7314)	9162 (7217)	9946 (6934)

Numbers in parentheses represent countries that have achieved fruit and vegetable availability greater than or equal to 400 g/person per day and their respective population. Food availability excludes consumer food waste. 1965 and 1990 values are taken from the FAOSTAT commodity balance sheets^27^ and 2015 and 2050 values are projections from the International Model for Policy Analysis of Agricultural Commodities and Trade using three scenarios (SSP 1–3) using default diets (based on historical trends). SSP=Shared Socioeconomic Pathway.

* The Baltic states are reported as part of the former Soviet Union up to 1990 and are included in Europe for all future projections.

**Figure 1:**
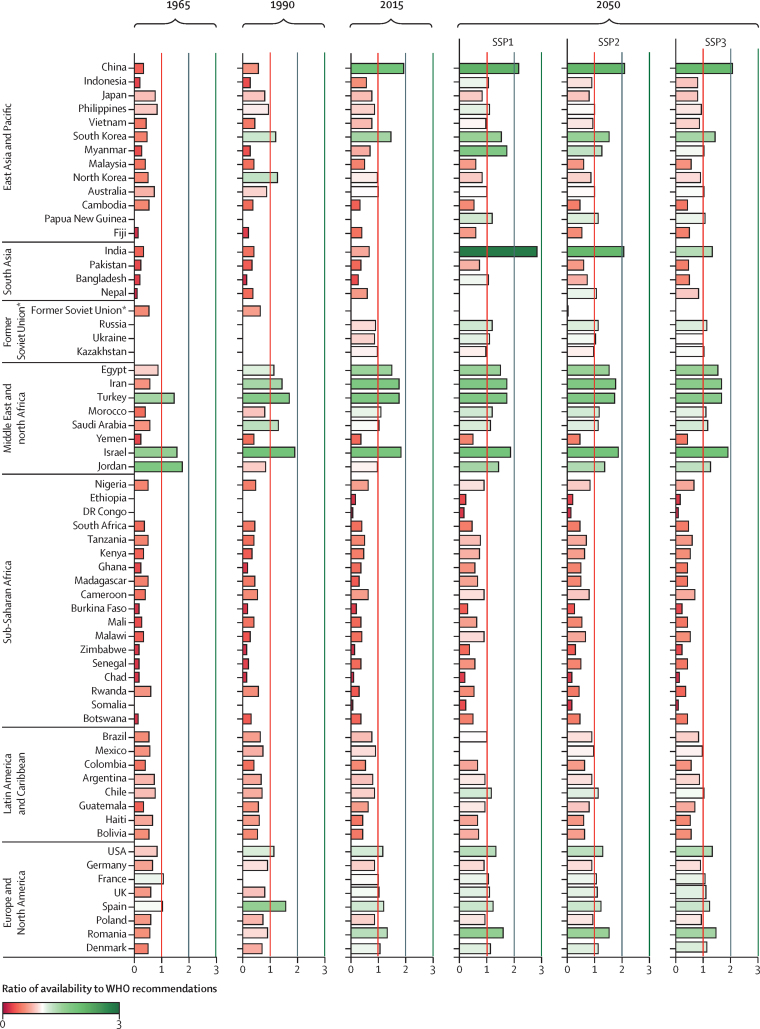
Selected country ratios of fruit and vegetable availability to WHO age-specific recommendations Countries shown were chosen to provide a representative group of countries from each region, including the maximum and minimum across the region. Countries in each region are listed in descending order by 2010 population. Food availability excludes consumer food waste. 1965 and 1990 values are from FAOSTAT commodity balance sheets^27^ and 2015 and 2050 values are projections from IMPACT using three scenarios (SSP1–3) using default diets (based on historical trends). Vegetables follow WHO definitions excluding legumes and starchy roots and tubers. The vertical lines represent when availability (excluding waste) equals one times (red), two times (grey), and three times (green) the population weighted average recommended consumption levels. IMPACT=International Model for Policy Analysis of Agricultural Commodities and Trade. SSP=Shared Socioeconomic Pathway. *Data up to 1990 are presented for the former Soviet Union as a whole, whereas data from 2015 onwards are presented separately for Russia, Ukraine, and Kazakhstan.

Progress has not been observed equally across all regions and many countries continue to have insufficient availability of fruits and vegetables to supply a healthy diet for all (figure 1). These numbers also do not take into account intracountry variation, where countries with sufficient availability often fail to achieve access for all within the country. High-income countries in Europe, North America, and the east Asia and Pacific region have made the most progress towards supplying sufficient fruits and vegetables over the past several decades. A substantial increase in availability has also been observed among the fast-growing economies of east and southeast Asia (eg, China, Vietnam, and South Korea). Economic development, although not equally distributed, has increased the resources available to billions of people, contributing to these improvements. Far less progress has been achieved in low-income countries in Asia and sub-Saharan Africa, which have experienced less economic development and more political instability (eg, DR Congo, Somalia, and Yemen)—one of the largest contributors to the recent uptick in the prevalence of hunger.^1^

Future economic growth will continue to be an important factor in determining global fruit and vegetable availability. In 2050, the global average fruit and vegetable availability varies from 608 g/person per day in SSP3 to 732 g in SSP2 and 862 g in SSP1 (table). Gains from future economic development are concentrated in developing regions, which are more sensitive to changes in economic growth assumptions. Several countries are projected to make substantial gains in fruit and vegetable availability by 2050, such as India and Indonesia (figure 1). However, many countries in Europe and the Americas fail to make considerable progress towards increasing the availability of fruits and vegetables across all three scenarios, despite being near the age-specific recommendations in 2015 (figure 1). Even more concerning is the number of countries in sub-Saharan Africa, parts of Asia, and the Pacific region that substantially fail to supply enough fruits and vegetables to meet recommended consumption levels by 2050 under the most optimistic economic projections. Sub-Saharan Africa is of particular concern: per-capita income growth in the region is projected between 2·2% per year under SSP3 and 4·7% per year under SSP1, but fewer than a third of the 43 countries in the region have sufficient fruit and vegetable availability to meet the 400 g/person per day minimum target in all three SSPs (table). With population in the region projected to reach between 1·5 billion and 2 billion people by 2050, between 0·8 billion and 1·9 billion people could live in countries with average fruit and vegetable availability less than 400 g/person per day (table); under the more stringent targets, between 1·4 billion and 2·0 billion people—essentially the whole region—could be living in countries with insufficient availability (figure 1).

**Figure 2:**
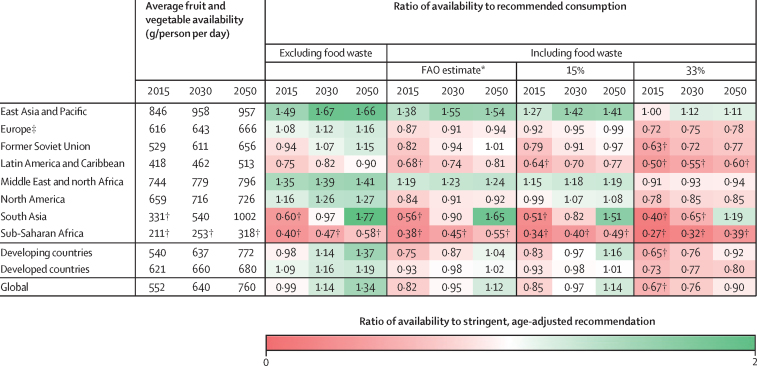
Regional summary of ratios of average fruit and vegetable availability to recommended consumption levels including and not including various levels of food waste Projections are considered under Shared Socioeconomic Pathway 2. Availability is population-weighted average for each region. Recommended consumption levels are 330–600 g/person per day, depending on age. FAO=Food and Agriculture Organization of the United Nations. *FAO waste estimates vary by region and range from 5% in sub-Saharan Africa to 28% in east Asia and Pacific and North America. †Fails to achieve the 400 g/person per day minimum recommendation. ‡Includes Baltic states.

When we adjust the projections highlighted in figure 1 to take into account food waste, we see the insufficiency of projected supply increase. Figure 2 presents aggregated regional results under SSP2 using recent FAO region-specific waste estimates.^31^ In 2015, this reduces the number of countries achieving an average fruit and vegetable supply of 400 g/person per day from 81 countries to 65 countries. This 16-country reduction represents a combined population of 0·7 billion people—nearly 10% of the global population. By 2030, assuming current waste levels remain unchanged, sub-Saharan Africa would fail to meet the 400 g/person per day threshold, with five additional regions (Europe, the former Soviet Union, Latin America and Caribbean, North America, and south Asia) failing to supply sufficient fruits and vegetables for the more stringent recommended consumption levels. By 2050, four regions would continue having average availability below recommended levels, with 22 fewer countries achieving this target, representing nearly 0·9 billion people living in countries with insufficient availability.

Under both alternative future waste assumptions (ie, 15% and 33%), we see erosions in the gains projected for many regions (figure 2). Assuming 33% waste, by 2050, average global supply fails to meet recommended consumption levels, with all but two regions—east Asia and Pacific and south Asia—falling below recommended levels and sub-Saharan Africa and Latin America and the Caribbean falling to less than 400 g/person per day on average. Under the 33% waste scenario, only 19 countries, representing 3·6 billion people would have sufficient fruit and vegetable availability to meet recommended consumption levels compared with 60 countries representing 5·1 billion people under the no waste scenario. Even under the more optimistic assumption of 15% waste, four regions would fall below recommended levels by 2050, with sub-Saharan Africa still supplying less than 400 g/person per day on average (figure 2). We show what the availability gap could look like assuming a more sustainable dietary pathway in the appendix (p 21), and even under this more sustainable dietary pathway many countries continue to supply insufficient fruits and vegetables on average.

**Figure 3:**
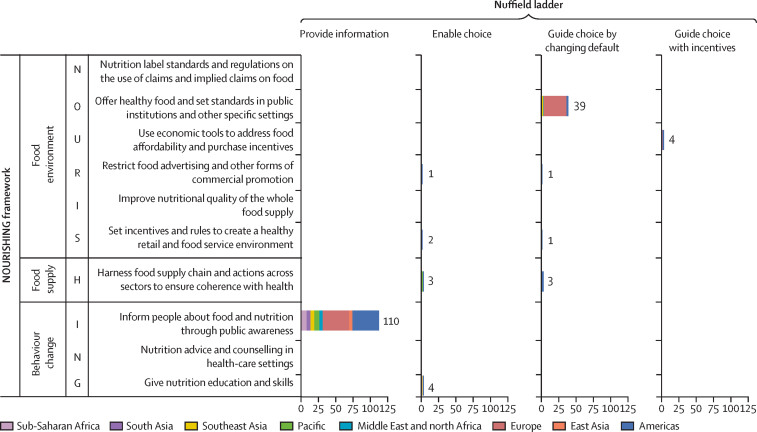
Summary of policy interventions in the NOURISHING database related to fruits and vegetables grouped by region and mapped to the Nuffield ladder Figure shows the number of countries for which the NOURISHING database describes either a minimum quantity of fruits and vegetables to be provided or some other activity that specifically promotes increased consumption of fruits or vegetables. No country examples were found for three Nuffield ladder policy options—guide choice with disincentives, restrict choice, and eliminate choice—which are therefore not presented on the figure. Some policies pertaining to general healthy eating, without specific mention of activities regarding promotion of increased consumption of fruits or vegetables, are excluded from this count. Regional policies are converted to country counts by assuming 28 countries are included in the EU, 15 countries in the Caribbean Community group, and 22 countries in Pacific Island Nations and Territories group.

Of the 764 policies in the World Cancer Research Fund International's NOURISHING database, only 168 specifically target fruit and vegetable consumption. Almost two thirds of these policies focused on behaviour change, most of which are food-based dietary guidelines, corresponding to the lowest or least forceful rung on the Nuffield ladder (figure 3). There were only 55 examples of policies targeting fruits and vegetables in the food environment (48 examples) and food supply (six examples), including examples from just four countries (Canada, Fiji, the UK, and the USA) of fiscal policies to reduce the cost of fruits and vegetables, corresponding to the more forceful rung on the Nuffield ladder of “guide choice through incentives” (figure 3). Furthermore, these national fiscal policies are often highly targeted and not comprehensive in coverage. For example, Canada's fiscal policies are subsidies targeted to increase access to healthy foods in isolated communities in northern Canada.^35^ This analysis highlights the relative dearth of policy options currently implemented to promote increased fruit and vegetable production and consumption.

## Discussion

Low fruit and vegetable consumption is an important and long-running challenge and has many inter-related causes, such as insufficient supply, poor access, low affordability, and high levels of waste.^40^ Reviewing historical data, we see there has been progress at increasing the availability of fruits and vegetables, although it has lagged behind progress made towards reducing hunger over the past several decades. Our results suggest we are likely to see continued progress but with substantial regional variation, with many regions failing to achieve adequate fruit and vegetable availability, hindering progress towards the second Sustainable Development Goal, Zero Hunger.

Our analysis builds on methods used in recent global assessments of the food system. We expand the analysis of food security using the SSPs by exploring in greater detail the impacts of projected agricultural production and demand on representative diets and compare them to WHO recommendations on healthy diets. This analysis consistently models the interaction of projected trends in population growth, economic development, agricultural productivity, and changes in consumer behaviour. To do this we have used IMPACT, an integrated economic model in combination with socioeconomic and waste scenarios. Global economic models such as IMPACT rely on aggregated national statistics (FAOSTAT) and simulate complex economic behaviour in fairly stylised ways. For example, IMPACT represents all production in terms of homogeneous commodities (eg, bananas instead of, say, Cavendish bananas) and simulates demand using a single representative consumer for each country. The global and national statistics used to build and calibrate these models are limited, with data often reported in aggregate (eg, citrus, other). Fruit and vegetable recommended consumption levels are also highly aggregated, with many cases not differentiating fruits and vegetables, and where there are disaggregated targets, they are fairly coarse (eg, leafy greens, orange vegetables). There is substantial variation on both the production and demand side within countries and commodity groups that global datasets and global models such as IMPACT cannot directly comment on. Downscaling the implications of global scenarios that inform general trends to better assess the specific impacts on different types of producers and consumers is an important area of future research.

We focused on the potential impacts of varying socioeconomic assumptions using the SSPs. The SSPs are long-run scenarios of the global economy and do not include other drivers that are important to global food security, such as extreme events, ecological collapse, state fragility, and transformative technologies. Climate change, which is also not included, is likely to lead to lower yields, higher food prices, and decreased availability of fruits and vegetables, particularly in lower-income countries.^16^ Nevertheless, changes in economic development^17^ and food policy^41^ are likely to have a greater impact than climate change up to the mid-21st century. The three scenarios we used provided a broad range of socioeconomic assumptions. However, there can be many representations of each SSP, which would affect the final results. Additionally, model selection incurs uncertainty in results. Ideally, this type of analysis could be completed using multi-model ensembles and various representations of the SSPs as has been done for land-use^14^ and climate change.^15^

The scarcity of global data on waste makes this a major point of uncertainty in trying to estimate average consumption globally. We elected to use a range of waste scenarios to give a sense of how far projected fruit and vegetable availability could be from average consumption. We recognise that this invariably means that the projected gap from these scenarios is somewhat crude. However, it serves to highlight the importance of increasing production of fruits and vegetables to ensure that supply is more than sufficient to satisfy recommended levels, as well as emphasising the need for further research on food waste and its impact on food value chains.

Economic development has been and will continue to be an important contributor to future progress. However, economic growth alone will be unlikely to lead to sufficient availability of fruits and vegetables in all regions, particularly when taking into account the effects of food waste. Globally, fruit and vegetable availability in 2050 under SSP1 could be more than 40% higher than under SSP3. This increase is driven almost entirely in lower-income regions such as sub-Saharan Africa and south Asia, with availability in developed countries substantially less sensitive to future economic growth assumptions. This suggests that policies that target poverty and broad economic growth might have important co-benefits in spurring increased demand and access to fruits and vegetables in lower-income countries. For higher-income countries, however, additional economic growth will probably have less of an impact, as economic constraints are less binding. Nevertheless, there is substantial variation within countries in terms of access and affordability with fruits and vegetables continuing to be relatively more expensive than other foods. Targeted policies on the consumption side, such as price subsidies, could increase the affordability of fruits and vegetables, leading to population-wide health benefits.^20^

Producing exactly the amount of fruits and vegetables required to satisfy WHO recommendations will ultimately be insufficient given the impact of food waste. However, we found that even with zero waste, many countries will need to increase their fruit and vegetable production to achieve adequate supply, a finding consistent with recent literature comparing production to dietary recommendations.^10^, ^11^ Increasing production will probably require prioritisation of investments in research and development around fruit and vegetable production, which has been relatively ignored.^42^ Given that most fruits and vegetables are used relatively near the point of production, these investments should be targeted to promote production in regions where projected supply is inadequate, such as sub-Saharan Africa, parts of Asia, and the Pacific. Investments in agriculture have been broadly shown to have substantial potential to increase incomes and food security, particularly in Africa.^43^ Therefore, investments in this sector could carry additional co-benefits in contributing to several Sustainable Development Goals (SDGs; eg, SDG 1: no poverty and SDG 2: zero hunger), especially when we consider the importance of small-scale producers in fruit and vegetable production in these regions.^44^

Approaches to increasing fruit and vegetable production must also be assessed on their potential environmental impacts. Fruit and vegetable production is more resource-intensive compared with other crops.^5^, ^6^ Increased consumption of fruits and vegetables could increase environmental pressure, unless it is accompanied by other shifts towards more sustainable production and consumption. Fruits and vegetables are also relatively perishable, with some estimates suggesting they contribute more than 40% of total food losses and waste.^30^ Food waste and food losses decrease consumption directly and indirectly by increasing the cost of fruits and vegetables. Under SSP2 and the high-waste scenario, we projected 139 countries, representing 5·6 billion people, with insufficient fruits and vegetables by 2050—an increase of 1·5 billion people compared with the no waste scenario. Efforts to reduce waste are crucial to achieving sustainable food systems but, if improperly targeted, could make fruits and vegetables more expensive. Ideally, policies could be coordinated to decrease unhealthy and resource-intensive components of modern diets while promoting healthy options. However, in practice, such coordination is challenging, which might lead to difficult trade-offs between environmental and health objectives in the short and medium term that are not fully taken into consideration in the discussion of win-win scenarios for environmental and health outcomes of diet changes.

Increasing the supply of fruits and vegetables is crucial to achieving recommended consumption levels; however, this is unlikely to be achieved without changing consumer behaviour, as low consumption continues even where availability is not a constraint. Public policies targeting consumer behaviour could help to shift consumption patterns. However, to date there has been little public policy activity in this space, and the activity that exists is primarily informational in nature. Provision of information is important and quantified food-based dietary guidelines provide valuable scientific targets. More countries need to develop them, particularly in Africa where only seven countries have done so.^24^ However, in isolation, informational policies are likely to be slow and ineffective at increasing fruit and vegetable consumption.^39^ For example, in the USA, 5 years after a 2007 update to fruit and vegetable consumption targets, only 6% of surveyed consumers were familiar with the new consumption targets and only an additional 30% were familiar with the previous targets established in 1991.^45^

Due to few policy interventions to date, it is difficult to know which policies could be most successful. However, lessons can be learned from policy interventions to reduce smoking, and current efforts to reduce fat and sugar consumption. These experiences would suggest that informational policies are more likely to be effective if supplemented by more forceful policy options along the Nuffield ladder that more actively and directly restructure the choice infrastructure within which both consumers and producers act, such as taxes and subsidies.

Increasing productivity of the fruit and vegetable sector could reduce the resource intensity of fruit and vegetable production, permitting more production at a lower cost to consumers and the environment. Reducing waste in fruit and vegetable value chains also can reduce the environmental footprint of their production and, if properly implemented and fully taking into account the benefits and costs,^33^ could save consumers money. Many of these interventions could be in the shape of investments in research and development to develop new and improved processing, storage and distribution technologies but could also include changes to labelling regulations (eg, use-by dates) and, more broadly, the promotion of new markets for by-products to encourage a more circular economy.

## Data sharing

Country-level results and interactive data visualisations used in this analysis are available at http://dx.doi.org/10.17632/d7m5h5zvw7.1.
