# Supplementary material for: Gaps between fruit and vegetable production, demand, and recommended consumption at global and national levels: an integrated modelling study
Source: Lancet Planet Health. 2019 Jul;3(7):e318–29. doi: 10.1016/S2542-5196(19)30095-6 (PMC6637854; doi:10.1016/S2542-5196(19)30095-6)
Supplement: Supplementary appendix [file mmc1.pdf]

THE LANCET
Planetary Health

Supplementary appendix

This appendix formed part of the original submission and has been peer reviewed. We post it as supplied by the authors.

Supplement to: Mason-D'Croz D, Bogard JR, Sulser TB, et al. Gaps between fruit and vegetable production, demand, and recommended consumption at global and national levels: an integrated modelling study. *Lancet Planet Health* 2019; **3:** 318–29.

Gaps between fruit and vegetable production, demand, and recommended consumption at global and national levels: An integrated modelling study

Supplementary Materials

Authors: Daniel Mason-D'Croz, Jessica R. Bogard, Timothy B. Sulser, Nicola Cenacchi, Shahnila Dunston, Mario Herrero, Keith Wiebe

Table of Contents

National and International Dietary Guidelines and Recommendations relating to Fruits and Vegetables 1
IMPACT Model Description and Relevant Commodity and Regional Definitions 2
Crop Production 2
Livestock Production 4
Production of Processed Goods 5
Commodity Demand 6
Markets, Trade, and Equilibrium Prices 8
Fruit and Vegetable Commodities in IMPACT 10
IMPACT Regional Definitions 10
Applying Food Waste in the Analysis 16
Scenario Assumptions 18
Socioeconomic assumptions in the SSPs 19
Additional Scenario Assumptions 20
Consumption preferences and sustainable diet 20
Agricultural productivity 20
Land-Use Regulation 20
Trade 20
Joining NOURISHING Framework and Nuffield Ladder 24
References 26

Figures

FIGURE S1 REPRESENTATION OF FOOD LOSSES AND WASTE IN IMPACT 16
FIGURE S2 RATIO OF PER CAPITA FRUIT & VEGETABLE AVAILABILITY TO WHO MINIMUM CONSUMPTION LEVEL (400 G/PERSON/DAY) UNDER DIFFERENT WASTE ASSUMPTIONS BY 2050 UNDER SSP2. 17
FIGURE S3 COMPARING THE RATIO OF FRUIT & VEGETABLE AVAILABILITY TO WHO MINIMUM RECOMMENDATION BY 2050 FOR SSP 1–3 UNDER DIFFERENT EXOGENOUS ASSUMPTIONS (DEFAULT AND SUSTAINABLE) ON FUTURE DIETARY TRANSITIONS 21
FIGURE S4 ALL CCOUNTRY RATIOS OF FRUIT & VEGETABLE AVAILABILITY TO WHO AGE-SPECIFIC RECOMMENDATIONS 22
FIGURE S5 ALL CCOUNTRY RATIOS OF FRUIT & VEGETABLE AVAILABILITY TO WHO MINIMUM RECOMMENDATION (400 G/PERSON/DAY) 23

Tables

TABLE S1 SUMMARY OF SELECTED NATIONAL AND INTERNATIONAL DIETARY GUIDELINES RELATING TO FRUIT AND VEGETABLES 1
TABLE S2 FRUIT AND VEGETABLE COMMODITIES IN IMPACT AND EQUIVALENT COMMODITIES IN FAOSTAT .. 10
TABLE S3 IMPACT COUNTRIES AND REGIONAL DEFINITIONS 10
TABLE S4 FAO REGIONAL FRUIT AND VEGETABLE WASTE ESTIMATES APPLIED TO IMPACT REGIONS 16
TABLE S5 SUMMARY OF SSP SCENARIO SPACE AND SCENARIO NARRATIVES 18
TABLE S6 SUMMARY OF SOCIOECONOMIC ASSUMPTIONS OF POPULATION, GDP, AND PER CAPITA GDP FOR SSP 1–3 19
TABLE S7 MAPPING THE NOURISHING FRAMEWORK TO THE NUFFIELD LADDER 25

National and International Dietary Guidelines and Recommendations relating to Fruits and Vegetables

Fruits and vegetables play a key role in promoting human health not only in their relative nutrient density, but also as important sources of fiber and other compounds associated with promoting healthier gut-biomes, strengthening the immune system, and reducing the risk of non-communicable diseases.[1] The recognition of the importance of eating fruits and vegetables and their "protective" role in a healthy diet is not new. The first formal dietary recommendation, issued in the United Kingdom in 1835, recognized the importance of citrus fruit to avoid scurvy and preceded the development of more complete dietary recommendations for energy and nutrient intake by more than a century.[2]

Today 87 countries have developed food-based dietary guidelines, in which almost all promote fruit and vegetable consumption, albeit with different definitions of what constitutes a fruit or vegetable, and varying recommended consumption levels.[3] WHO recommendations are consistent with national guidelines based on detailed health modelling. Table S1 highlights the similarity of national guidelines with the WHO recommendations.

Table S1 Summary of selected national and international dietary guidelines relating to fruit and vegetables

FAO Region	Country	Age group (years)	Servings Equivalent in grams<br>Vegetables	Fruits	Total
Africa and Middle East	Benin[4]	Adults and Children (2+)	100–750	75–450	175–1200
	Lebanon[5]	Adults and Children (2+)	250–450	300	550–750
	South Africa[6]	Adults and Children (4+)			320-400
Asia and Pacific	Australia[7]	Adults and Children (2+)	188–450	150-300	338–750
	China[8]	Adults and Children (2+)	300–500	200–350	500–850
	India[9]	Adults and Children (1+)	100-500	100	208–720
	Thailand[10]	Adults and Children (2+)	160–240	450–750	610–990
Europe and North America	Canada[11]	Adults and Children (2+)			400–1500
	Spain[12]	Adults and Children (2+)	300	360	660
	United States[13]	Adults and Children (2+)	250–450	300	550–750
Latin America And the Caribbean	Argentina[14]	Adults and Children (2+)	400	300	700
	Bolivia[15]	Adults and Children (6+)	100–600	200–400	300–1000
	Mexico[16]	Adults and Children (2+)	150–600	100–600	250–1200
International Recommendations	WHO[17,18]	Minimum Recommendation<br>Adults and Children			400<br>330-600

Color Legend:	Vegetables incl. starchy roots and pulses	Vegetables incl. starchy roots, pulses unspecified	Vegetables incl. starchy roots, excl. pulses	Vegetables excl. starchy roots and pulses

Note: Where dietary guidelines didn't specify servings in grams, serving definitions were matched to Australia's dietary guidelines and estimated in grams

The WHO recommendations are also broadly consistent with the benchmark healthy diet presented in the Eat-Lancet commission study on healthy diets from sustainable food systems, falling within the range of healthy consumption levels (300-900 g/person/day). Globally the age-specific WHO recommendations would suggest global average fruit and vegetable availability around 560 g/person/day, quite similar to the median EAT-Lancet recommendation of 500 g/person/day of fruits and vegetables (300 g/person/day of vegetables and 200 g/person/day of fruits) for adults and children 2+ years of age.[19]

IMPACT Model Description and Relevant Commodity and Regional Definitions

In this analysis we used the quantified scenarios from IMPACT from the latest round of work from the Agricultural Intercomparison and Improvement Project (AgMIP).[20,21] IMPACT is a global economic model that was developed at IFPRI in the 1990s to contribute to foresight efforts around the 2020 Vision initiative[22], and to address a lack of foresight tools to inform policymakers and researchers on the policies that would be necessary to ensure global food security into the future. Over time this economic model has been continuously improved, linking the core economic model to biophysical models (climate, crop, hydrology, etc.), environmental, health, and a range of complimentary economic models.

At the core of this expanding integrated modelling system is a multi-market partial equilibrium model of the global agriculture sector. IMPACT simulates global production, trade, prices, and demand for 62 agricultural commodities in 158 countries and regions. The multi-market model simulates national and global agricultural markets, solving for equilibrium prices and quantities, such that global demand (including waste and losses) equals global supply. IMPACT simulates agricultural markets from 2005 to 2050, with the initial 10 years (2005-2015) serving as a calibration period to adjust to historical data.

The following description is drawn from the full model documentation[23] which is available at: http://ebrary.ifpri.org/cdm/ref/collection/p15738coll2/id/129825.

Crop Production

Crop production in IMPACT is simulated through area[1] and yield response functions. The choice of specifying crop production in this way has a long history in IMPACT and facilitates interaction with commodity experts and land-use specialists, who work in natural units (hectares, tons per hectare). Crop production in IMPACT is specified sub-nationally with the area and yield functions at the level of FPUs. This regional disaggregation permits linking with water models and provides the added benefit of smaller geographical units for aggregating climate change results, which can vary significantly from one location to another. Land used for crop production is divided into irrigated and rainfed systems, capturing the significant differences in yields observed across these cultivation systems and linking directly with the water models, which treat irrigated and rainfed water supplies separately.

IMPACT uses a land market to manage competing demands for agricultural land from different crops, as well as providing new linkage points to land-use models that work with broader land-use changes, such as conversion of forest to grasslands and agricultural land. It also allows us to separate total area supply (irrigated and rainfed) from individual crop area demands and allows equilibrium conditions to determine the best economic use of the available land. The total supply of land is assumed to be a function of the scarcity value or shadow price index of land, which can also be considered a summary of changes in crop prices. The shadow price (WF) is indexed to 1 in the first year and changes based on changing demands from all crops for land area.

$$
\begin{aligned}
QFS_{\mathrm{fpu,lnd}} &= QFSInt_{\mathrm{fpu,lnd}} \times QFSInt2_{\mathrm{fpu,lnd}} \\
QFS &= \text{Land supply} \\
QFSInt &= \text{Land supply intercept (base year supply)} \\
QFSInt2 &= \text{Land supply growth multiplier} \\
fpu &= \text{Food production unit} \\
lnd &= \text{Land type (i.e. irrigated, rainfed)}
\end{aligned}
$$

The supply of land is considered exogenous within each year, meaning that farmers are not allowed to adjust the total crop area in the middle of the year. The total land supply over time is driven by exogenous trends on the availability of area for agriculture as well as endogenous responses to changes in area demand, which is handled in between years. The following equation is applied at the end of each year before solving for a new year.

[1] In IMPACT, area is treated as harvested area, which is the total area planted and harvested within a year, and may include multi-cropping or multiple harvests and differ from total arable land or reported physical area.

$$QFSInt2_{\text{fpu,lnd,t+1}} = QFSInt2_{\text{fpu,lnd,t}} \times \left(1 + Landgr_{\text{fpu,lnd}}\right) \times \left(\frac{WF_{\text{fpu,lnd,t}}}{\left\langle WF_{\text{fpu,lnd,t}} \right\rangle_{t-3}} \right)^{L\gamma}$$

$Landgr$ = Exogenous land supply growth rate

$\left\langle WF_{\text{fpu,lnd,t}} \right\rangle_{t-3}$ = Average shadow price of past 3 years

$L\gamma$ = Land supply elasticity

Crop area is specified as an area demand function with respect to changes in the marginal revenue product, changes in land cost, and exogenous non-price trends in harvested area. Crop area elasticities simulate the supply response to changes in the marginal revenue of land represented by the following equation as the interaction of the net price of an activity and the productivity of the activity in using an additional hectare of land.

$$MRP_{\text{j,fpu,lnd}} = Yld_{\text{j,fpu,lnd}} \times PNET_{\text{j,cty}}$$

MRP = Marginal revenue product of land

Yld = Crop yield

$PNET$ = Net price for the activity at the country-level mapped to fpu

j = Activity (crop)

cty = Country

The exogenous trend in harvested area captures changes in area resulting from factors other than direct market effects, such as government programs encouraging cropping expansion, contraction due to soil degradation, or conversion of land from agriculture to non-agricultural uses. The combination of these endogenous and exogenous factors in area demand are described in the following equation.

$$Area_{\text{j,fpu,lnd}} = AreaInt_{\text{j,fpu,lnd}} \times AreaInt2_{\text{j,fpu,lnd}} \times WF_{\text{fpu,lnd}}^{WF\varepsilon} \times \left(\frac{MRP_{\text{j,fpu}}}{MRP0_{\text{j,fpu}}} \right)^{A\varepsilon}$$

$Area$ = Final crop area

$AreaInt$ = Crop area intercept (base year crop area)

$AreaInt2$ = Exogenous crop area growth multiplier

$WF\varepsilon$ = Elasticity of demand with respect to land shadow price

$MRP0$ = Base year marginal revenue product (used to index prices)

$A\varepsilon$ = Elasticity of area demand with respect to marginal revenue product

Assumptions for exogenous trends are determined by a combination of historical changes in land use and expert judgment on potential future regional dynamics. They are represented as compound growth from the base and are applied between years.

$$AreaInt2_{\text{j,fpu,lnd,t+1}} = AreaInt2_{\text{j,fpu,lnd,t}} \times \left(1 + Areagr_{\text{fpu,lnd}}\right)$$

$Areagr$ = Exogenous area demand growth rate

Competing demands from different crops are handled through an equilibrium equation that determines the land allocation and ensures that all crop area demand sums up to the total land supply for each FPU.

$$QFS_{fpu,lnd} = \sum_{j} Area_{j,fpu,lnd}$$

Crop yields are a function of commodity prices, prices of inputs, available water, climate, and exogenous trend factors. The IMPACT model includes five ways that changes in yields are achieved. First, the model assumes a scenario of underlying improvements in yields over time that, to varying degrees, continue trends observed during the past 50 to 60 years in an informed extrapolation. These long-run trends, or intrinsic productivity growth rates, are intended to reflect the expected increases in inputs, improved seeds, and improvements in management practices. These trends differ and generally are higher for developing countries, where there is considerable scope to narrow the gap in yields compared to developed countries. These intrinsic productivity growth rates are exogenous to the model, and changes in them are specified as part of the definition of different scenarios. We assume that these underlying trends vary by crop and region and that they will decline somewhat

during the next 50 years as the pace of technological improvements in developed countries slows and as developing countries catch up to yields in developed countries.

Second, the IMPACT model includes a short-run (annual), endogenous, response of yields to changes in both input and output prices. These yield response functions specify the change in yield as a constant elasticity function of the changes in output prices, with elasticity parameters that can vary by crop and region. The underlying assumption is that farmers will respond to changes in prices by varying the use of inputs, including inputs such as fertilizer, chemicals, and labor that will, in turn, change yields.

Third, climate is assumed to affect yields through two mechanisms. The first is through the effects of changes in temperature and weather due to climate change on crop yields for rainfed and irrigated crops, as calculated from the solution of a crop simulation model for different climate change scenarios. These crop simulations vary by crop type. The crop models are run with detailed time, geographic, and crop disaggregation for different climate change scenarios that are downscaled to include weather variation in small geographic areas. This analysis gives changes in average yields due to climate change that are then averaged to generate yield shocks by crop and region. The second mechanism by which climate change affects yields is through variation in water availability for agriculture year by year in different climate scenarios. This mechanism is modeled through the use of the IMPACT water models. These include (1) a global hydrology model that determines runoff to the river basins included in the IMPACT model; (2) water basin management models for each FPU that optimally allocate available water to competing non-agricultural and agricultural uses, including irrigation; and (3) a water allocation and stress model that allocates available irrigation water to crops and, when the water supply is less than demand by crop, computes the impact of the water shortage on crop yields, accounting for differences among crops and varieties. These yields shocks are then passed to the IMPACT model, affecting year-to-year crop yields.

$$Yield_{\text{j,fpu,lnd}} = YieldInt_{\text{j,fpu,lnd}} \times YieldInt2_{\text{j,fpu,lnd}} \times WatShk_{\text{j,fpu,lnd}} \times CliShk_{\text{j,fpu,lnd}} \times \left(\frac{PNET_{\text{j,cty}}}{PNET0_{\text{j,cty}}} \right)^{Y\varepsilon} \times PF^{F\varepsilon}$$

$Yield$ = Final yield

$YieldInt$ = Yield intercept (base year yield)

$YieldInt2$ = Exogenous yield growth multiplier

$WatShk$ = Water stress shock (from water models)

$CliShk$ = Climate change shock (from water and crop models)

$Y\varepsilon$ = Yield supply elasticity with respect to net price

PF = Input prices

$F\varepsilon$ = Yield supply elasticity with respect to input prices

Final crop production for each FPU and crop (j) is estimated as the product of the solution for its respective area and yield equations, with national production ($QS_{\text{j,cty}}$) equal to the summation of the production in all of the relevant FPUs in that country.

$$QS_{\text{j,cty}} = \sum_{\text{fpu,lnd}} \left(Area_{\text{j,fpu,lnd}} \times Yield_{\text{j,fpu,lnd}} \right)$$

Livestock Production

Livestock production is modeled at the FPU level and includes animal numbers, with associated feed demands, and meat/dairy production based on processing the animals. Similar to the crop sector, this specification allows for easier translation of information from livestock experts who are used to working with herd-size and feeding requirements. In the current version of the model, there is no modeling of herd dynamics—herd size over time is set exogenously.

Feed demand is a function of the livestock's own price, the prices of intermediate (feed) inputs, and a trend variable reflecting growth in livestock herds (slaughter rates are implicitly assumed to stay more or less constant over time). The price elasticities in the livestock supply function are derived in a fashion similar to how the crop area and yield elasticities are derived.

$$Animals_{\text{j,fpu,livsys}} = AnimalInt_{\text{j,fpu,livsys}} \times AnimalInt2_{\text{j,fpu,livsys}} \times \left(\frac{PNET_{\text{j,cty}}}{PNET0_{\text{j,cty}}}\right)^{AN\varepsilon} \times \prod_{\text{cfeeds}} \left(\frac{PC_{\text{c,cty}}}{PC0_{\text{c,cty}}}\right)^{Feed\varepsilon}$$

$Animals$ = Number of producing animals
$AnimalInt$ = Animal intercept (initial number of animals)
$AnimalInt2$ = Exogenous population growth
PC = Consumer prices
$PC0$ = Intial consumer prices
$Feed\varepsilon$ = Supply elasticity with respect to changes in feed prices
$livsys$ = Livestock production systems
$cfeeds$ = Feed commodities demanded by livestock sector

Livestock yields are determined through exogenous growth due to improved animals and management practices. Currently, all price responses in the livestock sector are accounted for in the animal number equations.

$$AnimalYield_{\text{j,fpu,livsys}} = AnimalYieldInt_{\text{j,fpu,livsys}} \times AnimalYieldInt2_{\text{j,fpu,livsys}}$$

$AnimalYield$ = Animal yields
$AnimalYieldInt$ = Initial animal yields
$AnimalYieldInt2$ = Exogenous yield growth

Total national production ($QS_{\text{j,cty}}$) is calculated by multiplying the number of slaughtered animals by the yield per head and summing across FPU and livestock system.

$$QS_{\text{j,cty}} = \sum_{\text{fpu,livsys}} \left(Animals_{\text{j,fpu,livsys}} \times AnimalYield_{\text{j,fpu,livsys}}\right)$$

There is work under way to improve the livestock model, incorporating more animal types; a number of feed systems that include pastures, fodders, processed feeds, and feed grains; and a more detailed representation of the value chain from feeds to herds to final demand commodities.

Production of Processed Goods

Modelling of processed goods (that is, food oils, oil meals, sugar) has been an active area of improvement for IMPACT 3, and the development of the activity-commodity framework allows for a general handling of all processed goods in IMPACT through input-output matrixes and the use of net prices. The input-output matrixes represent technical coefficients on input requirements, are specified by quantities of inputs per unit of output (that is, metric tons of soybeans per metric tons of soybean oil), and are calculated from the base data. The net price is the price the producer receives net of input costs. The net price will equal the producer price of the activity whenever there are no intermediate inputs.

$$PNET_{\text{j,cty}} = PP_{\text{j,cty}} - \sum_{\text{inputs}} \left(IOMAT_{\text{inputs,j,cty}} \times \left(1 - CSEI_{\text{inputs,cty}}\right) \times PC_{\text{inputs,cty}}\right)$$

$PNET$ = Net price
PP = Producer price
PC = Consumer price of inputs
$CSEI$ = Consumer support estimate on intermediate inputs
$IOMAT$ = Input-output matrix
$inputs$ = Set of commodities (c) that are inputs into activity j

Production of processed goods is then simulated by a supply function that incorporates both endogenous price effects and exogenous technological change. As opposed to crop and livestock production, processed goods are modeled at the country level instead of at the FPU.

$$QS_{\mathrm{j,cty}} = QSInt_{\mathrm{j,cty}} \times QSINT2_{\mathrm{j,cty}} \times \left(\frac{PNET_{\mathrm{j,cty}}}{PNET0_{\mathrm{j,cty}}} \right)^{QS\varepsilon}$$

$QS =$ Total production
$QSInt =$ Initial production
$QSInt2 =$ Exogenous productivity growth
$QS\varepsilon =$ Supply elasticity with respect to net price

Commodity Demand

Total domestic demand for a commodity is the sum of household food demand, agricultural intermediate demand (feed and processed goods), and intermediate demand from other sectors (that is, for biofuels and industrial uses).

$$QD_{\mathrm{c,cty}} = \sum_{\mathrm{h}} \left(QH_{\mathrm{c,h,cty}} \right) + QInterm_{\mathrm{c,cty}} + QL_{\mathrm{c,cty}} + QBF_{\mathrm{c,cty}} + QOTH_{\mathrm{c,cty}}$$

$QD =$ Total commodity demand
$QH =$ Household food demand
$QInterm =$ Intermediate demand from Ag-processing sector
$QL =$ Feed demand from livestock sector
$QBF =$ Intermediate demand for biofuel feedstock
$QOth =$ All other demand
$h =$ Household type

Food demand is a function of the price of the commodity and the prices of other competing commodities, per capita income, and total population. Per capita income and population increase annually according to country-specific population and income growth rates. Population and gross domestic product (GDP) trends vary by scenario and are drawn from the Shared Socio-economic Pathway (SSP) describes in a later section. The IMPACT demand elasticities are estimated and adjusted to represent a synthesis of average, aggregate elasticities for each region, given the income level and distribution of urban and rural population. Over time the elasticities are adjusted to accommodate the gradual shift in demand from staples to high-value commodities like meat, especially in developing countries. This assumption is based on expected economic growth, increased urbanization, and continued commercialization of the agricultural sector. IMPACT is designed to simulate multiple types of households (that is, rural, urban, rich, poor, and so forth); however, currently, IMPACT treats household demand with one representative consumer per country.

$$QH_{c,h,cty} = QHInt_{c,h,cty} \times \left(\frac{pcGDP_{h,cty}}{pcGDP0_{h,cty}} \right)^{Inc\varepsilon} \times \left(\frac{(1-CSE_{c,cty}) \times PC_{c,cty}}{(1-CSE0_{c,cty}) \times PC0_{c,cty}} \right)^{HF\varepsilon}$$

$$\times \prod_{cc \neq c} \left(\frac{(1-CSE_{cc,cty}) \times PC_{cc,cty}}{(1-CSE0_{cc,cty}) \times PC0_{cc,cty}} \right)^{HF\varepsilon} \times \frac{PopH_{h,cty}}{PopH0_{h,cty}}$$

QH = Household food demand

$QHInt$ = Initial household food demand

$pcGDP$ = Per capita GDP

$pcGDP0$ = Initial per capita GDP

CSE = Consumer support estimate

$CSE0$ = Initial consumer support estimate

$PopH$ = Population disaggregated by household type

$PopH0$ = Initial household population

$Inc\varepsilon$ = Income demand elasticity

$HF\varepsilon$ = Price demand elasticty

$\left(\frac{(1-CSE) \times PC}{(1-CSE0) \times PC0} \right)^{HF\varepsilon}$ = Own-price response

$\prod_{cc \neq c} \left(\frac{(1-CSE) \times PC}{(1-CSE0) \times PC0} \right)^{HF\varepsilon}$ = Cross-price response

Feed demand is a derived intermediate demand. It is determined by two components: (1) animal feed requirements determined by livestock production and livestock feed requirements and (2) price effects that take into account potential substitution possibilities among different feeds. The equation also incorporates a technology parameter that indicates improvements in feeding efficiencies over time.

$$QL_{c,cty} = \sum_{jlvst} \left(QS_{jlvst,cty} \times Req_{jlvst,c,cty} \right) \times \prod_{cfeeds} \left(\frac{PC_{c,cty}}{PC0_{c,cty}} \right)^{LFD\varepsilon}$$

QL = Total feed demand for livestock sector

QS = Total production of each livestock activity

Req = Feed requirements for each livestock activity

$LFD\varepsilon$ = Price elasticity of demand for feed

$jlvst$ = Set of livestock producing activities

Intermediate demand is a derived demand that is based on the demand for final processed goods, such as food oils and sugar. The input-output matrix determines the proportions of inputs (c) required for each producing activity (j).

$$QDInterm_{c,cty} = \sum_{j} \left(IOMat_{c,j,cty} \times QS_{j,cty} \right)$$

$QDInterm$ = Intermediate demand

$IOMat$ = Input-Output matrix

Exogenous biofuel feedstock demand is determined through exogenous growth rates, which represent government mandates to encourage the production of biofuels, though adjusted in various scenarios where the mandates are infeasible or adjusted to reflect scenarios on the role of first- or second-generation biofuels. The biofuel feedstock demand equation also allows for a price response for biofuels to allow for substitution across different potential feedstocks as well as to reflect the reality that increasing food prices would put pressure to ease biofuel mandates.

$$QBF_{c,cty} = QBFInt_{c,cty} \times QBFINT2_{c,cty} \times \prod_{c} \left(\frac{PC_{c,cty}}{PC0_{c,cty}} \right)^{BF\varepsilon}$$

QBF = Biofuel feedstock demand

$QBFInt$ = Initial demand from biofuel sector

$QBFInt2$ = Exogenous growth in demand from biofuels

$BF\varepsilon$ = Price elasticity of demand for biofuel feedstock

Other demand summarizes all other demands for agricultural products from sectors outside of the focus of IMPACT (for example, seeds, industrial use, and waste). It is simulated under two equations. The primary method follows the household food demand equation and is sensitive to changes in income, population, and prices.

$$QOth_{c,cty} = QOthInt_{c,cty} \times \left(\frac{pcGDP_{cty}}{pcGDP0_{cty}} \right)^{IOth\varepsilon} \times \left(\frac{POP_{cty}}{POP0_{cty}} \right) \times \prod_{cc} \left(\frac{PC_{c,cty}}{PC0_{c,cty}} \right)^{POth\varepsilon}$$

$QOth$ = Other Demand

$QOthInt$ = Initial other demand

$IOth\varepsilon$ = Income demand elasticity for other demand

$POth\varepsilon$ = Price demand elasticity for other demand

The second method is used in a few cases where other demand historically has not shown much of a response to prices and is instead a function of changes in per capita GDP from the previous year (*pcGDP*1).

$$QOth_{c,cty} = QOth1_{c,cty} \times \frac{pcGDP_{cty}}{pcGDP1_{cty}}$$

$QOth$ = Other demand

$QOth1$ = Lagged other demand

$pcGDP1$ = Lagged per capita GDP

Markets, Trade, and Equilibrium Prices

The system of equations finds a set of domestic and world prices for all crops that clear domestic and international commodity markets. The world price of a commodity is the equilibrating mechanism for traded commodities—when an exogenous shock is introduced in the model, world price will adjust to clear world markets, and each adjustment is passed back to the effective producer and consumer prices via the price transmission equations. Changes in domestic prices subsequently affect commodity supply and demand, necessitating their iterative readjustments until world supply and demand balance and world net trade again equals 0. For non-traded commodities, domestic prices in each country adjust to equate supply and demand within the country.

IMPACT assumes a closed world economy—at the end of every year the world's production must equal the world's demand. This constraint is ensured by the following equation, where the sum of net trade over the globe must equal 0.

$$\sum_{cty} NT_{c,cty} = 0$$

NT = Net Trade

National production and demand for tradable commodities are linked to world markets through trade. Commodity trade by country (cty) is a function of domestic production, domestic demand, and stock change. Regions with positive net trade are net exporters, while those with negative values are net importers. This specification does not permit a separate identification of international trade by country of origin and destination—all countries export to and import from a single global market.

$$NT_{c,cty} = QSUP_{c,cty} - QD_{c,cty} - QSt_{c,cty}$$

NT = Net trade

QSt = Change in stocks

Prices are endogenous in the system of equations for food and are calibrated to 2005 commodity prices, are reported in constant 2005 US dollars. Domestic prices of tradable commodities are a function of world prices, adjusted by the effect of trade policy represented by taxes and tariffs, and price policies are expressed in terms of producer support estimates (PSEs), consumer support estimates (CSEs), and the cost of moving products from one market to another represented by marketing margins (MMs). Export taxes and import tariffs are also included to allow the representation of national trade policies and their impacts on agricultural markets. MMs reflect other factors such as transport and marketing costs of getting goods to various markets and are based on expert opinion on the quality and availability of transportation, communication, and market infrastructure.

The model includes three markets: (1) the farm gate, where producers sell their output to purchasers in producer prices; (2) a national market, where the purchasers then take the commodity, incurring any taxes/subsidies and trade/transportation costs; and (3) the port where exports are sold to foreigners and imports are bought from them at world market prices. Moving commodities to and from the port incurs MMs and any taxes/subsidies/tariffs. In the model, PSEs, CSEs, and MMs are expressed as percentages (ad valorem) of the world price. To calculate producer prices the appropriate wedges are applied to the domestic consumer prices (*PC*) and represent the markup observed in domestic markets from the farm-gate or factory-gate prices producers receive. The producer price of an activity is the weighted sum of the prices of the commodities associated with that activity.

$$PP_{\text{j,cty}} \times \left(1 + MMJ_{\text{j,cty}}\right) = \left(1 + PSE_{\text{j,cty}}\right) \times \sum_{\text{c}} JCRatio_{\text{j,c,cty}} \times PC_{\text{c,cty}}$$

PP = Producer price

MMJ = Farm(factory)-gate to domestic market Marketing Margin (MM)

PSE = Producer support estimate, ad valorem component

$JCRatio$ = mapping from activities (j) to commodities (c)

How consumer prices are determined in IMPACT depends on the state of tradability of the commodity. Commodities can be specified as either tradable or non-tradable. Traded commodity prices are determined in international markets. Non-traded commodities are those commodities whose prices are determined in national markets, without direct links to international markets. Examples include sugarcane, sugar beets, and grass, where all demand is intermediate demand from domestic sectors (sugar processing and livestock). These commodity prices are determined endogenously by country and ensure that domestic supply equals domestic demand.

$$QSUP_{\text{c,cty}} = QD_{\text{c,cty}}$$

Non-traded commodities are indirectly linked to world markets through the demand for final products (that is, sugar), and potential substitution from tradable commodities (that is, grass and other feeds). IMPACT 3 also has been designed to allow the tradability of a commodity to be determined endogenously. As the IMPACT model includes price wedges between domestic and international markets, the prices of exports received by producers and of imports paid by consumers can be modeled in separate equations.

$$PM_{\text{c,cty}} = PW_{\text{c}} \times EXR_{\text{cty}} \times \left(1 + TM_{\text{c,cty}}\right) \times \left(1 + MMM_{\text{c,cty}}\right)$$

$$PE_{\text{c,cty}} = PW_{\text{c}} \times EXR_{\text{cty}} \times \left(1 - TE_{\text{c,cty}}\right) \times \left(1 - MME_{\text{c,cty}}\right)$$

PM = Import Price

PE = Export Price

PW = World Price

EXR = Exchange Rate (currently =1)

TM = Import tariff (ad valorem)

TE = Export tax (ad valorem)

MMM = Marketing margin for importing to domestic market

MME = Marketing margin for exporting to international market

If the equilibrium domestic price falls between the floor price of exports and the ceiling price of imports, then there will be no international trade. If conditions change (over time or for different scenarios) such that the equilibrium domestic price either falls to the export price or rises to the import price, the model will endogenously change the regime and clear the market through international trade. To start importing the

domestic import price must equal the consumer price (global prices are lower than domestic prices), and to start exporting domestic prices must be equal to export prices (domestic prices are greater than global prices).

$$\text{Imports if } PC_{c,cty} \leq PM_{c,cty}$$
$$\text{Exports if } PC_{c,cty} \geq PE_{c,cty}$$
$$\text{Domestically traded if } PE_{c,cty} \leq PC_{c,cty} \leq PM_{c,cty}$$

For purely tradable goods, where we want the commodities to always be linked to world markets, this inequality is not used, the domestic consumer price is set to the import price, and the export price equation is never used.

$$PC_{c,cty} = PM_{c,cty}$$

Fruit and Vegetable Commodities in IMPACT

Of IMPACT's 62 simulated agricultural commodities four are relevant to the fruit and vegetable discussion. Table S2 summarizes these commodities, and what items from FAOSTAT are represented within them.

Table S2 Fruit and Vegetable commodities in IMPACT and equivalent commodities in FAOSTAT

IMPACT code	IMPACT name	FAO name	FAO Code
jbana	Bananas	Bananas	2615
jsubf	(Sub)-Tropical Fruits	Oranges, Mandarins	2611
		Lemons, Limes	2612
		Grapefruit	2613
		Citrus, Other	2614
		Pineapples	2618
		Dates	2619
jtemf	Temperate Fruits	Apples	2617
		Grapes	2620
		Fruit, other	2625
		Fruits - Excluding Wine	2919
jvege	Vegetables	Tomatoes	2601
		Onions	2602
		Vegetables, Other	2605
		Pepper	2640
		Pimento	2641

Source: Robinson et al.[23]

IMPACT Regional Definitions

IMPACT simulates agricultural production, trade and demand globally and nationally with a high level of regional disaggregation for a global model. IMPACT simulates agricultural markets in 158 countries, which we have grouped into 9 geographic regions and 2 income groups (developed and developing) for reporting and analysis purposes. Table S3 presents IMPACT countries and regions, along with the regions in which they have been grouped for this analysis.

Table S3 IMPACT Countries and Regional Definitions

IMPACT Code	IMPACT Name	ISO Code	ISO Name	Region Code(s)	Region(s)
AFG	Afghanistan	AFG	Afghanistan	SAS, DVG, WLD	South Asia, Developing Countries, World
AGO	Angola	AGO	Angola	SSA, DVG, WLD	Sub-Saharan Africa, Developing Countries, World
ALB	Albania	ALB	Albania	EUR, DVG, WLD	Europe, Developing Countries, World
ARG	Argentina	ARG	Argentina	LAC, DVG, WLD	Latin America and Caribbean, Developing Countries, World
ARM	Armenia	ARM	Armenia	FSU, DVG, WLD	Former Soviet Union, Developing Countries, World
AUS	Australia	AUS	Australia	EAP, DVD, WLD	East Asia and Pacific, Developed Countries, World

IMPACT Code	IMPACT Name	ISO Code	ISO Name	Region Code(s)	Region(s)
AUT	Austria	AUT	Austria	EUR, DVD, WLD	Europe, Developed Countries, World
AZE	Azerbaijan	AZE	Azerbaijan	FSU, DVG, WLD	Former Soviet Union, Developing Countries, World
BDI	Burundi	BDI	Burundi	SSA, DVG, WLD	Sub-Saharan Africa, Developing Countries, World
BEN	Benin	BEN	Benin	SSA, DVG, WLD	Sub-Saharan Africa, Developing Countries, World
BFA	Burkina Faso	BFA	Burkina Faso	SSA, DVG, WLD	Sub-Saharan Africa, Developing Countries, World
BGD	Bangladesh	BGD	Bangladesh	SAS, DVG, WLD	South Asia, Developing Countries, World
BGR	Bulgaria	BGR	Bulgaria	EUR, DVG, WLD	Europe, Developing Countries, World
BLR	Belarus	BLR	Belarus	FSU, DVG, WLD	Former Soviet Union, Developing Countries, World
BLT	Baltic States	EST	Estonia	EUR, DVD, WLD	Europe, Developed Countries, World
		LTU	Lithuania		
		LVA	Latvia		
BLX	Belgium-Luxembourg	BEL	Belgium	EUR, DVD, WLD	Europe, Developed Countries, World
		LUX	Luxembourg		
BLZ	Belize	BLZ	Belize	LAC, DVG, WLD	Latin America and Caribbean, Developing Countries, World
BOL	Bolivia	BOL	Bolivia	LAC, DVG, WLD	Latin America and Caribbean, Developing Countries, World
BRA	Brazil	BRA	Brazil	LAC, DVG, WLD	Latin America and Caribbean, Developing Countries, World
BTN	Bhutan	BTN	Bhutan	SAS, DVG, WLD	South Asia, Developing Countries, World
BWA	Botswana	BWA	Botswana	SSA, DVG, WLD	Sub-Saharan Africa, Developing Countries, World
CAF	Central African Republic	CAF	Central African Republic	SSA, DVG, WLD	Sub-Saharan Africa, Developing Countries, World
CAN	Canada	CAN	Canada	NAM, DVD, WLD	North America, Developed Countries, World
CHL	Chile	CHL	Chile	LAC, DVG, WLD	Latin America and Caribbean, Developing Countries, World
CHM	China Plus	CHN	China	EAP, DVG, WLD	East Asia and Pacific, Developing Countries, World
		HKG	Hong Kong		
		MAC	Macao		
		TWN	Taiwan		
CHP	Switzerland Plus	CHE	Switzerland	EUR, DVD, WLD	Europe, Developed Countries, World
		LIE	Liechtenstein		
CIV	Ivory Coast	CIV	Ivory Coast	SSA, DVG, WLD	Sub-Saharan Africa, Developing Countries, World
CMR	Cameroon	CMR	Cameroon	SSA, DVG, WLD	Sub-Saharan Africa, Developing Countries, World
COD	Democratic Republic of Congo	COD	Democratic Republic of Congo	SSA, DVG, WLD	Sub-Saharan Africa, Developing Countries, World
COG	Congo	COG	Congo	SSA, DVG, WLD	Sub-Saharan Africa, Developing Countries, World
COL	Colombia	COL	Colombia	LAC, DVG, WLD	Latin America and Caribbean, Developing Countries, World
CRI	Costa Rica	CRI	Costa Rica	LAC, DVG, WLD	Latin America and Caribbean, Developing Countries, World
CUB	Cuba	CUB	Cuba	LAC, DVG, WLD	Latin America and Caribbean, Developing Countries, World
CYP	Cyprus	CYP	Cyprus	EUR, DVD, WLD	Europe, Developed Countries, World
CZE	Czech Republic	CZE	Czech Republic	EUR, DVD, WLD	Europe, Developed Countries, World
DEU	Germany	DEU	Germany	EUR, DVD, WLD	Europe, Developed Countries, World
DJI	Djibouti	DJI	Djibouti	SSA, DVG, WLD	Sub-Saharan Africa, Developing Countries, World
DNK	Denmark	DNK	Denmark	EUR, DVD, WLD	Europe, Developed Countries, World
DOM	Dominican Republic	DOM	Dominican Republic	LAC, DVG, WLD	Latin America and Caribbean, Developing Countries, World
DZA	Algeria	DZA	Algeria	MEN, DVG, WLD	Middle East and North Africa, Developing Countries, World
ECU	Ecuador	ECU	Ecuador	LAC, DVG, WLD	Latin America and Caribbean, Developing Countries, World

IMPACT Code	IMPACT Name	ISO Code	ISO Name	Region Code(s)	Region(s)
EGY	Egypt	EGY	Egypt	MEN, DVG, WLD	Middle East and North Africa, Developing Countries, World
ERI	Eritrea	ERI	Eritrea	SSA, DVG, WLD	Sub-Saharan Africa, Developing Countries, World
ETH	Ethiopia	ETH	Ethiopia	SSA, DVG, WLD	Sub-Saharan Africa, Developing Countries, World
FJI	Fiji	FJI	Fiji	EAP, DVG, WLD	East Asia and Pacific, Developing Countries, World
FNP	Finland Plus	ALA	Aland Islands	EUR, DVD, WLD	Europe, Developed Countries, World
		FIN	Finland		
FRP	France Plus	FRA	France	EUR, DVD, WLD	Europe, Developed Countries, World
		MCO	Monaco		
GAB	Gabon	GAB	Gabon	SSA, DVG, WLD	Sub-Saharan Africa, Developing Countries, World
GEO	Georgia	GEO	Georgia	FSU, DVG, WLD	Former Soviet Union, Developing Countries, World
GHA	Ghana	GHA	Ghana	SSA, DVG, WLD	Sub-Saharan Africa, Developing Countries, World
GIN	Guinea	GIN	Guinea	SSA, DVG, WLD	Sub-Saharan Africa, Developing Countries, World
GMB	Gambia	GMB	Gambia	SSA, DVG, WLD	Sub-Saharan Africa, Developing Countries, World
GNB	Guinea-Bissau	GNB	Guinea-Bissau	SSA, DVG, WLD	Sub-Saharan Africa, Developing Countries, World
GNQ	Equatorial Guinea	GNQ	Equatorial Guinea	SSA, DVG, WLD	Sub-Saharan Africa, Developing Countries, World
GRC	Greece	GRC	Greece	EUR, DVD, WLD	Europe, Developed Countries, World
GRL	Greenland	GRL	Greenland	NAM, DVD, WLD	North America, Developed Countries, World
GSA	Guyanas South America	GUF	French Guiana	LAC, DVG, WLD	Latin America and Caribbean, Developing Countries, World
		GUY	Guyana		
		SUR	Suriname		
GTM	Guatemala	GTM	Guatemala	LAC, DVG, WLD	Latin America and Caribbean, Developing Countries, World
HND	Honduras	HND	Honduras	LAC, DVG, WLD	Latin America and Caribbean, Developing Countries, World
HRV	Croatia	HRV	Croatia	EUR, DVD, WLD	Europe, Developed Countries, World
HTI	Haiti	HTI	Haiti	LAC, DVG, WLD	Latin America and Caribbean, Developing Countries, World
HUN	Hungary	HUN	Hungary	EUR, DVD, WLD	Europe, Developed Countries, World
IDN	Indonesia	IDN	Indonesia	EAP, DVG, WLD	East Asia and Pacific, Developing Countries, World
IND	India	IND	India	SAS, DVG, WLD	South Asia, Developing Countries, World
IRL	Ireland	IRL	Ireland	EUR, DVD, WLD	Europe, Developed Countries, World
IRN	Iran	IRN	Iran	MEN, DVG, WLD	Middle East and North Africa, Developing Countries, World
IRQ	Iraq	IRQ	Iraq	MEN, DVG, WLD	Middle East and North Africa, Developing Countries, World
ISL	Iceland	ISL	Iceland	EUR, DVD, WLD	Europe, Developed Countries, World
ISR	Israel	ISR	Israel	MEN, DVD, WLD	Middle East and North Africa, Developed Countries, World
ITP	Italy Plus	ITA	Italy	EUR, DVD, WLD	Europe, Developed Countries, World
		MLT	Malta		
		SMR	San Marino		
		VAT	Vatican City		
JAM	Jamaica	JAM	Jamaica	LAC, DVG, WLD	Latin America and Caribbean, Developing Countries, World
JOR	Jordan	JOR	Jordan	MEN, DVG, WLD	Middle East and North Africa, Developing Countries, World
JPN	Japan	JPN	Japan	EAP, DVD, WLD	East Asia and Pacific, Developed Countries, World
KAZ	Kazakhstan	KAZ	Kazakhstan	FSU, DVG, WLD	Former Soviet Union, Developing Countries, World
KEN	Kenya	KEN	Kenya	SSA, DVG, WLD	Sub-Saharan Africa, Developing Countries, World
KGZ	Kyrgyzstan	KGZ	Kyrgyzstan	FSU, DVG, WLD	Former Soviet Union, Developing Countries, World
KHM	Cambodia	KHM	Cambodia	EAP, DVG, WLD	East Asia and Pacific, Developing Countries, World
KOR	South Korea	KOR	South Korea	EAP, DVD, WLD	East Asia and Pacific, Developed Countries, World

IMPACT Code	IMPACT Name	ISO Code	ISO Name	Region Code(s)	Region(s)
LAO	Laos	LAO	Laos	EAP, DVG, WLD	East Asia and Pacific, Developing Countries, World
LBN	Lebanon	LBN	Lebanon	MEN, DVG, WLD	Middle East and North Africa, Developing Countries, World
LBR	Liberia	LBR	Liberia	SSA, DVG, WLD	Sub-Saharan Africa, Developing Countries, World
LBY	Libya	LBY	Libya	MEN, DVG, WLD	Middle East and North Africa, Developing Countries, World
LKA	Sri Lanka	LKA	Sri Lanka	SAS, DVG, WLD	South Asia, Developing Countries, World
LSO	Lesotho	LSO	Lesotho	SSA, DVG, WLD	Sub-Saharan Africa, Developing Countries, World
MDA	Moldova	MDA	Moldova	FSU, DVG, WLD	Former Soviet Union, Developing Countries, World
MDG	Madagascar	MDG	Madagascar	SSA, DVG, WLD	Sub-Saharan Africa, Developing Countries, World
MEX	Mexico	MEX	Mexico	LAC, DVG, WLD	Latin America and Caribbean, Developing Countries, World
MLI	Mali	MLI	Mali	SSA, DVG, WLD	Sub-Saharan Africa, Developing Countries, World
MMR	Myanmar	MMR	Myanmar	EAP, DVG, WLD	East Asia and Pacific, Developing Countries, World
MNG	Mongolia	MNG	Mongolia	EAP, DVG, WLD	East Asia and Pacific, Developing Countries, World
MOR	Morocco Plus	ESH	Western Sahara	MEN, DVG, WLD	Middle East and North Africa, Developing Countries, World
		MAR	Morocco		
MOZ	Mozambique	MOZ	Mozambique	SSA, DVG, WLD	Sub-Saharan Africa, Developing Countries, World
MRT	Mauritania	MRT	Mauritania	MEN, DVG, WLD	Middle East and North Africa, Developing Countries, World
MWI	Malawi	MWI	Malawi	SSA, DVG, WLD	Sub-Saharan Africa, Developing Countries, World
MYS	Malaysia	MYS	Malaysia	EAP, DVG, WLD	East Asia and Pacific, Developing Countries, World
NAM	Namibia	NAM	Namibia	SSA, DVG, WLD	Sub-Saharan Africa, Developing Countries, World
NER	Niger	NER	Niger	SSA, DVG, WLD	Sub-Saharan Africa, Developing Countries, World
NGA	Nigeria	NGA	Nigeria	SSA, DVG, WLD	Sub-Saharan Africa, Developing Countries, World
NIC	Nicaragua	NIC	Nicaragua	LAC, DVG, WLD	Latin America and Caribbean, Developing Countries, World
NLD	Netherlands	NLD	Netherlands	EUR, DVD, WLD	Europe, Developed Countries, World
NOR	Norway	NOR	Norway	EUR, DVD, WLD	Europe, Developed Countries, World
NPL	Nepal	NPL	Nepal	SAS, DVG, WLD	South Asia, Developing Countries, World
NZL	New Zealand	NZL	New Zealand	EAP, DVD, WLD	East Asia and Pacific, Developed Countries, World
OBN	Other Balkans	BIH	Bosnia-Herzegovina	EUR, DVD, WLD	Europe, Developed Countries, World
		MKD	Macedonia (FYR)		
		MNE	Montenegro		
		SRB	Serbia		
OSA	Other Southeast Asia	BRN	Brunei	EAP, DVG, WLD	East Asia and Pacific, Developing Countries, World
		SGP	Singapore		
PAK	Pakistan	PAK	Pakistan	SAS, DVG, WLD	South Asia, Developing Countries, World
PAN	Panama	PAN	Panama	LAC, DVG, WLD	Latin America and Caribbean, Developing Countries, World
PER	Peru	PER	Peru	LAC, DVG, WLD	Latin America and Caribbean, Developing Countries, World
PHL	Philippines	PHL	Philippines	EAP, DVG, WLD	East Asia and Pacific, Developing Countries, World
PNG	Papua New Guinea	PNG	Papua New Guinea	EAP, DVG, WLD	East Asia and Pacific, Developing Countries, World
POL	Poland	POL	Poland	EUR, DVD, WLD	Europe, Developed Countries, World
PRK	North Korea	PRK	North Korea	EAP, DVG, WLD	East Asia and Pacific, Developing Countries, World
PRT	Portugal	PRT	Portugal	EUR, DVD, WLD	Europe, Developed Countries, World
PRY	Paraguay	PRY	Paraguay	LAC, DVG, WLD	Latin America and Caribbean, Developing Countries, World
PSE	Occupied Palestinian Territory	PSE	Occupied Palestinian Territory	MEN, DVG, WLD	Middle East and North Africa, Developing Countries, World

IMPACT Code	IMPACT Name	ISO Code	ISO Name	Region Code(s)	Region(s)
RAP	Rest of Arab Peninsula	ARE	United Arab Emirates	MEN, DVD, WLD	Middle East and North Africa, Developed Countries, World
		BHR	Bahrain		
		KWT	Kuwait		
		OMN	Oman		
		QAT	Qatar		
ROU	Romania	ROU	Romania	EUR, DVG, WLD	Europe, Developing Countries, World
RUS	Russia	RUS	Russia	FSU, DVG, WLD	Former Soviet Union, Developing Countries, World
RWA	Rwanda	RWA	Rwanda	SSA, DVG, WLD	Sub-Saharan Africa, Developing Countries, World
SAU	Saudi Arabia	SAU	Saudi Arabia	MEN, DVD, WLD	Middle East and North Africa, Developed Countries, World
SDN	Sudan Plus	SDN	Sudan	SSA, DVG, WLD	Sub-Saharan Africa, Developing Countries, World
		SSD	South Sudan		
SEN	Senegal	SEN	Senegal	SSA, DVG, WLD	Sub-Saharan Africa, Developing Countries, World
SLB	Solomon Islands	SLB	Solomon Islands	EAP, DVG, WLD	East Asia and Pacific, Developing Countries, World
SLE	Sierra Leone	SLE	Sierra Leone	SSA, DVG, WLD	Sub-Saharan Africa, Developing Countries, World
SLV	El Salvador	SLV	El Salvador	LAC, DVG, WLD	Latin America and Caribbean, Developing Countries, World
SOM	Somalia	SOM	Somalia	SSA, DVG, WLD	Sub-Saharan Africa, Developing Countries, World
SPP	Spain Plus	AND	Andorra	EUR, DVD, WLD	Europe, Developed Countries, World
		ESP	Spain		
		GIB	Gibraltar		
SVK	Slovakia	SVK	Slovakia	EUR, DVD, WLD	Europe, Developed Countries, World
SVN	Slovenia	SVN	Slovenia	EUR, DVD, WLD	Europe, Developed Countries, World
SWE	Sweden	SWE	Sweden	EUR, DVD, WLD	Europe, Developed Countries, World
SWZ	Swaziland	SWZ	Swaziland	SSA, DVG, WLD	Sub-Saharan Africa, Developing Countries, World
SYR	Syria	SYR	Syria	MEN, DVG, WLD	Middle East and North Africa, Developing Countries, World
TCD	Chad	TCD	Chad	SSA, DVG, WLD	Sub-Saharan Africa, Developing Countries, World
TGO	Togo	TGO	Togo	SSA, DVG, WLD	Sub-Saharan Africa, Developing Countries, World
THA	Thailand	THA	Thailand	EAP, DVG, WLD	East Asia and Pacific, Developing Countries, World
TJK	Tajikistan	TJK	Tajikistan	FSU, DVG, WLD	Former Soviet Union, Developing Countries, World
TKM	Turkmenistan	TKM	Turkmenistan	FSU, DVG, WLD	Former Soviet Union, Developing Countries, World
TLS	Timor-L'Este	TLS	Timor-L'Este	EAP, DVG, WLD	East Asia and Pacific, Developing Countries, World
TUN	Tunisia	TUN	Tunisia	MEN, DVG, WLD	Middle East and North Africa, Developing Countries, World
TUR	Turkey	TUR	Turkey	MEN, DVG, WLD	Middle East and North Africa, Developing Countries, World
TZA	Tanzania	TZA	Tanzania	SSA, DVG, WLD	Sub-Saharan Africa, Developing Countries, World
UGA	Uganda	UGA	Uganda	SSA, DVG, WLD	Sub-Saharan Africa, Developing Countries, World
UKP	Great Britain Plus	GBR	Great Britain	EUR, DVD, WLD	Europe, Developed Countries, World
		GGY	Guernsey		
		IMN	Isle of Man		
		JEY	Jersey		
UKR	Ukraine	UKR	Ukraine	FSU, DVG, WLD	Former Soviet Union, Developing Countries, World
URY	Uruguay	URY	Uruguay	LAC, DVG, WLD	Latin America and Caribbean, Developing Countries, World
USA	United States	USA	United States	NAM, DVD, WLD	North America, Developed Countries, World
UZB	Uzbekistan	UZB	Uzbekistan	FSU, DVG, WLD	Former Soviet Union, Developing Countries, World
VEN	Venezuela	VEN	Venezuela	LAC, DVG, WLD	Latin America and Caribbean, Developing Countries, World
VNM	Vietnam	VNM	Vietnam	EAP, DVG, WLD	East Asia and Pacific, Developing Countries, World
VUT	Vanuatu	VUT	Vanuatu	EAP, DVG, WLD	East Asia and Pacific, Developing Countries, World

IMPACT Code	IMPACT Name	ISO Code	ISO Name	Region Code(s)	Region(s)
YEM	Yemen	YEM	Yemen	MEN, DVG, WLD	Middle East and North Africa, Developing Countries, World
ZAF	South Africa	ZAF	South Africa	SSA, DVG, WLD	Sub-Saharan Africa, Developing Countries, World
ZMB	Zambia	ZMB	Zambia	SSA, DVG, WLD	Sub-Saharan Africa, Developing Countries, World
ZWE	Zimbabwe	ZWE	Zimbabwe	SSA, DVG, WLD	Sub-Saharan Africa, Developing Countries, World

Source: Robinson et al.[23]

Applying Food Waste in the Analysis

Food waste is a potential factor in failing to achieving recommended consumption levels of fruits and vegetables. Waste can be defined in a variety of ways. IMPACT reflects this by accounting for Food Losses and Waste at different stages of the food value chain. Initial losses at the point of primary production (crops, animal) are represented in IMPACT as lower crop and animal yields. IMPACT has been used in several studies considering the effects of reducing these harvest and post-harvest losses.[24] Food losses and waste also happen in the processing, and distribution of food commodities from the farm-gate to the market where consumers can access them. These losses are reflected in the FAO commodity balance sheet as waste, and are simulated in IMPACT as a part of Other Demand.[23,25] Recent studies using IMPACT have considered the potential of reducing this waste in efforts to fully quantify SSP 1 and the sustainable development pathway represented by the scenario narrative.[20,21] Consumer waste is not endogenous captured in IMPACT, but is applied post-solution, when trying to convert food availability results from IMPACT into estimated average consumption in recent studies looking at diets and health outcomes.[26,27] Figure S1 summarizes how waste is represented in IMPACT.

Figure S1 Representation of Food Losses and Waste in IMPACT

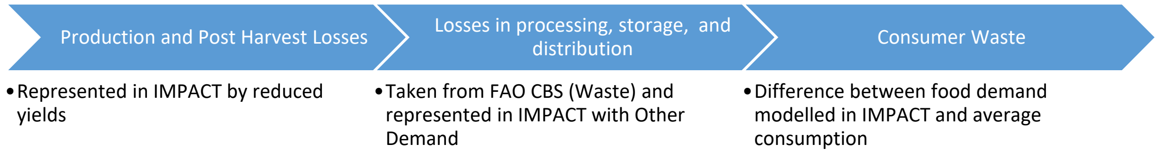

In this analysis we use regional waste estimated by Gustavsson et al.[28] Table S4 summarizes how we applied these regional estimates to IMPACT's geography.

Table S4 FAO Regional Fruit and Vegetable Waste Estimates Applied to IMPACT regions

IMPACT Region	Waste Estimate (%)
Developed Asian Economies	15
East, South and Southeast Asia	7
Oceania	28
Europe	19
Former Soviet Union	12
Middle East and North Africa	12
Sub-Saharan Africa	5
Latin America and Caribbean	10
North America	28

Source: Waste estimates are drawn from Gustavsson et al.[28] for fruits and vegetables and mapped to the appropriate IMPACT region. IMPACT regional definitions are found in Table S3

Figure S2 summarizes IMPACT projections by 2050 under the SSP 2 scenario. The figure presented the projected ratio of per capita fruit and vegetable availability compared to the minimum target (400 g/person/day) under a range of waste assumptions ranging from no waste to 33 percent waste. These are the disaggregated country results reflected in the regional aggregated results presented in Figure 2.

Summarizes IMPACT projections

Figure S2 Ratio of per capita fruit & vegetable availability to WHO minimum consumption level (400 g/person/day) under different waste assumptions by 2050 under SSP2.

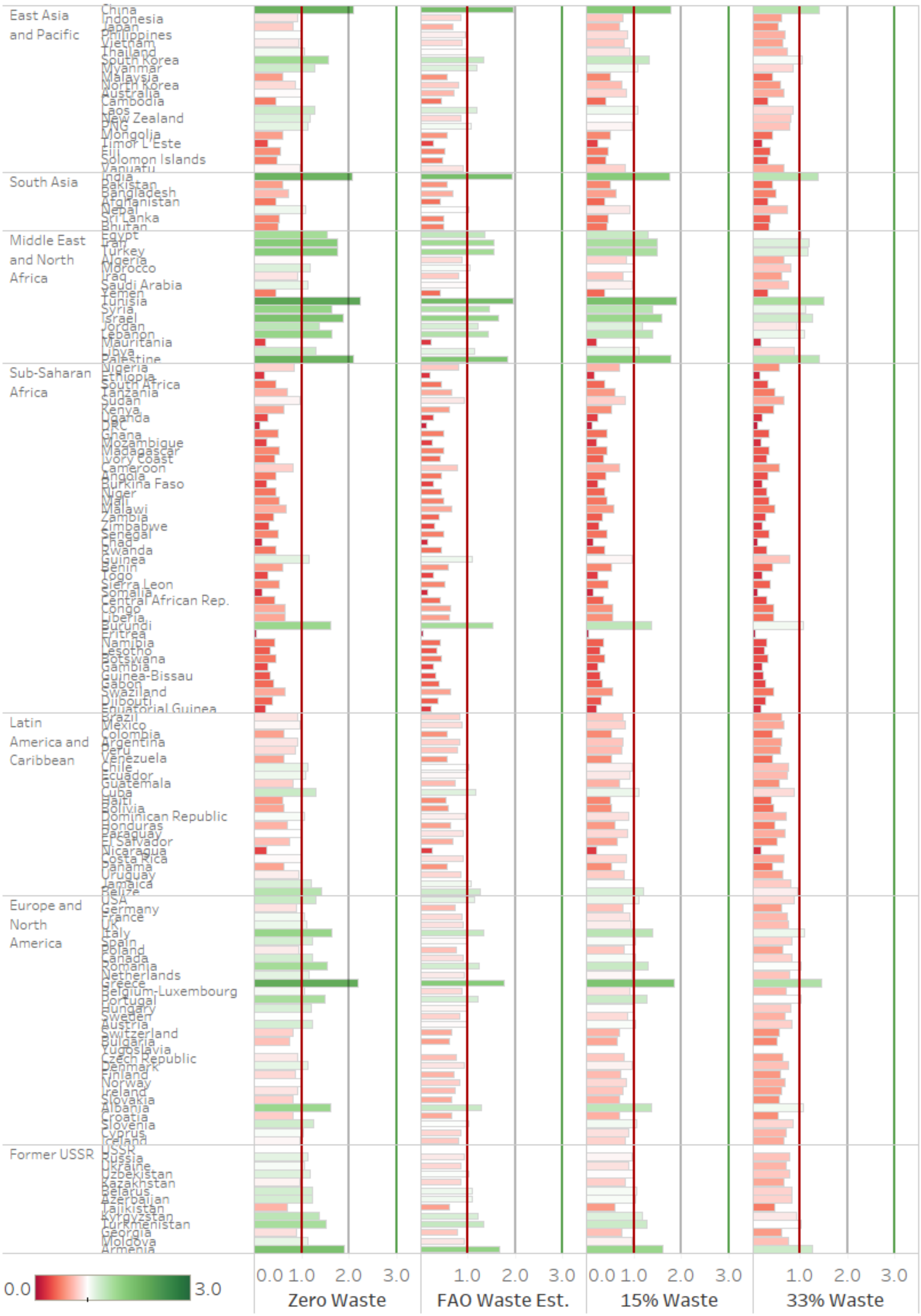

Note: FAO estimate by Gustavsson et al.[28] Each bar represents a country, with countries sorted in descending order by 2010 population. SSP 2 2050 values are projections from IMPACT using default diets (based on historical trends), and vegetables follow WHO definitions excluding legumes, and starchy roots and tubers. The vertical red, gray, and green lines represents when availability (excl. waste) equals 400, 800, and 1200 g/person/day respectively

Scenario Assumptions

The shared socioeconomic pathways (SSPs) are a set of five global scenarios developed for work by the International Panel for Climate Change (IPCC). They were designed to reflect a range of alternative futures where societal developments presented future challenges to climate change mitigation and adaptation. In this analysis we selected the three scenarios that go from low challenges to both mitigation and adaption (SSP 1) to high challenges to both mitigation and adaptation (SSP 3). Table S5 represents the axis upon which the SSPs were developed, along with the scenario narratives for the three selected SSPs used in this analysis.

Table S5 Summary of SSP scenario space and scenario narratives

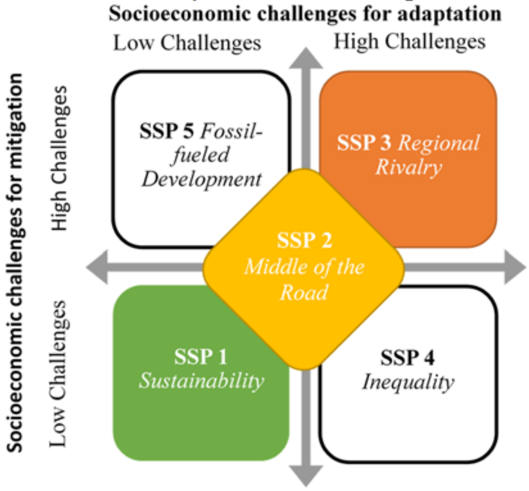

SSP 2 "Middle of the Road"

The world follows a path in which social, economic, and technological trends do not shift markedly from historical patterns. Development and income growth proceeds unevenly, with some countries making relatively good progress while others fall short of expectations. Global and national institutions work toward but make slow progress in achieving sustainable development goals. Environmental systems experience degradation, although there are some improvements and overall the intensity of resource and energy use declines. Global population growth is moderate and levels off in the second half of the century. Income inequality persists or improves only slowly and challenges to reducing vulnerability to societal and environmental changes remain.

SSP1 "Sustainability – Taking the Green Road"

The world shifts gradually, but pervasively, toward a more sustainable path, emphasizing more inclusive development that respects perceived environmental boundaries. Management of the global commons slowly improves, educational and health investments accelerate the demographic transition, and the emphasis on economic growth shifts toward a broader emphasis on human well-being. Driven by an increasing commitment to achieving development goals, inequality is reduced both across and within countries. Consumption is oriented toward low material growth and lower resource and energy intensity.

SSP 3 "Regional Rivalry – A Rocky Road"

A resurgent nationalism, concerns about competitiveness and security, and regional conflicts push countries to increasingly focus on domestic or, at most, regional issues. Policies shift over time to become increasingly oriented toward national and regional security issues. Countries focus on achieving energy and food security goals within their own regions at the expense of broader-based development. Investments in education and technological development decline. Economic development is slow, consumption is material-intensive, and inequalities persist or worsen over time. Population growth is low in industrialized and high in developing countries. A low international priority for addressing environmental concerns leads to strong environmental degradation in some regions.

Source: Figure based on Figure 1 in O'Neill et al.[29]; Scenario descriptions from Riahi et al.[30]

Socioeconomic assumptions in the SSPs

Table S6 summarizes the quantified socioeconomic assumptions by region and globally for SSP 1–3, and highlights the broad possibility space reflected by these three scenarios with respect to population and economic growth.

Table S6 Summary of Socioeconomic assumptions of population, GDP, and per capita GDP for SSP 1–3

	2010	2050			Average annual growth rate (% per year)		
**Region**		**SSP 1**	**SSP 2**	**SSP 3**	**SSP 1**	**SSP 2**	**SSP 3**
**East Asia and the Pacific**							
GDP	19,236	104,096	80,045	60,608	4.31	3.63	2.91
Population	2,184	2,173	2,261	2,351	–0.01	0.09	0.18
GDP per capita	9	48	35	26	4.32	3.54	2.72
**Europe**							
GDP	14,628	30,571	27,780	21,342	1.86	1.62	0.95
Population	537	592	577	498	0.24	0.18	–0.19
GDP per capita	27	52	48	43	1.61	1.43	1.14
**Former Soviet Union (excluding Baltic states)**							
GDP	2,855	10,603	8,984	7,551	3.33	2.91	2.46
Population	279	262	277	289	–0.15	–0.01	0.09
GDP per capita	10	40	32	26	3.5	2.92	2.37
**Latin America and the Caribbean**							
GDP	5,834	22,838	19,164	15,894	3.47	3.02	2.54
Population	585	674	742	853	0.36	0.6	0.95
GDP per capita	10	34	26	19	3.1	2.41	1.57
**Middle East and North Africa**							
GDP	4,551	20,566	18,631	16,006	3.84	3.59	3.19
Population	457	646	715	808	0.87	1.13	1.43
GDP per capita	10	32	26	20	2.95	2.43	1.73
**North America**							
GDP	14,290	33,691	29,933	24,753	2.17	1.87	1.38
Population	344	460	450	372	0.73	0.67	0.19
GDP per capita	41	73	67	67	1.43	1.19	1.19
**South Asia**							
GDP	4,461	44,250	32,939	22,756	5.9	5.13	4.16
Population	1,630	2,108	2,373	2,720	0.65	0.94	1.29
GDP per capita	3	21	14	8	5.23	4.14	2.83
**Sub-Saharan Africa**							
GDP	1,705	19,690	13,962	9,665	6.31	5.4	4.43
Population	863	1,564	1,793	2,084	1.5	1.84	2.23
GDP per capita	2	13	8	5	4.74	3.49	2.16
**World**							
GDP	67,559	286,305	231,439	178,575	3.68	3.13	2.46
Population	6,879	8,479	9,187	9,975	0.52	0.73	0.93
GDP per capita	10	34	25	18	3.14	2.38	1.51

Source: Robinson et al.[23], calculated from IMPACT 3 base year population and GDP with population and GDP growth rates from KC and Lutz,[31] and Dellink et al.[30], downloaded from SSP Database[32].
Note: Population is in millions. GDP (billion 2005 USD) and GDP per capita (000 USD/person) are in purchasing power parity. IMPACT regional definitions are found in Table S3

Additional Scenario Assumptions

The SSPs were quantified in IMPACT beyond just the socioeconomic drivers to incorporate scenario representation of other key components of the scenario narrative including consumption preferences, agricultural productivity, land-use regulation, and trade. This work was done for as inputs to recent AgMIP analysis[20,21], and is briefly summarized here.

Consumption preferences and sustainable diet

The SSP storylines describe differences in consumer behavior towards environmental impact of food consumption via food waste and share of animal products in the diet. For SSP 1, the sustainable diet was represented as a reduction in animal sourced food demand and an increase in plant-based diets. This sustainable dietary pathway is generally consistent with that outlined by Willet et al.[19] In the representation of SSP 1 in Hasegawa et al.[20] this sustainable diet was assumed. However, in this analysis we used scenarios where we continued using IMPACT's default diet based on historical trends (CONS2), with the understanding that achieving the sustainable dietary pathway will require more efforts in the policy space than currently observed. The scenario representation of SSP 1 with default diet was quantified as a part of a decomposition exercise to better understand the impacts of different scenario assumptions detailed in work by Stehfest et al.[21] For comparison purposes, Figure S3 shows the results in 2050 under SSP 1–3 using both the default assumption used in this study as well as the exogenous sustainable diet assumption.

Agricultural productivity

The SSP storylines describe fast technological change and respect for environmental boundaries for SSP1, and slower technological change in agricultural systems in SSP3. We adjust agricultural productivity based on the relationships between GDP, agricultural R&D and investment levels, and relationships between agricultural R&D and agricultural productivity. These relationships are described in more detail in Rosegrant et al.[33] and Mason-D'Croz et al.[34]

Land-Use Regulation

Land-use regulation was varied across the SSPs, and were implemented in IMPACT as exogenous assumptions on the availability of future land to be converted to agricultural uses, with land expansion curtailed under SSP 1 as compared to SSP 2. SSP 3 mirrored this treatment, with additional land allowed to be converted to agricultural uses.

Trade

Trade in agricultural commodities is effected by trade policy as describing in the market equilibrium and trade section above. To reflect varying levels of openness to global trade in the scenario narratives, current trade barriers were reduced in SSP 1 as compared to SSP 2, which uses current trade policies. Similar to land-use, in SSP 3 trade barriers were increased.

Figure S3 Comparing the ratio of fruit & vegetable availability to WHO minimum recommendation by 2050 for SSP 1–3 under different exogenous assumptions (default and sustainable) on future dietary transitions

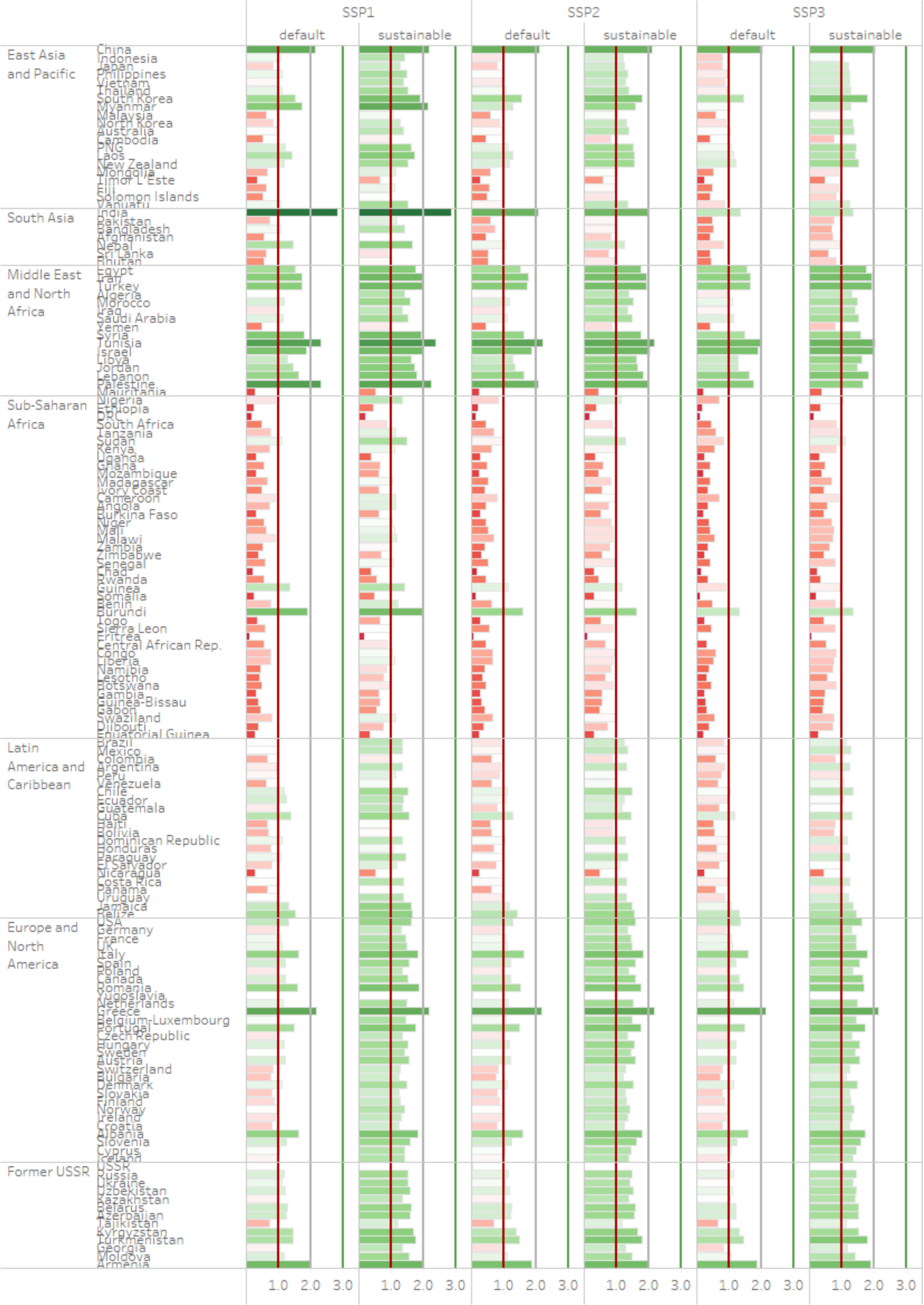

Notes: Countries sorted in descending order by 2010 population. Food availability excludes consumer food waste. 1965-2015 values are taken from FAOSTAT Commodity Balance Sheets[35], and 2050 values are projections from IMPACT using 3 scenarios (SSP 1–3) using default diets (based on historical trends), and vegetables follow WHO definitions excluding legumes, and starchy roots and tubers. The vertical red, gray, and green lines represents when availability (excl. waste) equals 400, 800, and 1200 g/person/day respectively

Figure S4 All ccountry ratios of fruit & vegetable availability to WHO age-specific recommendations

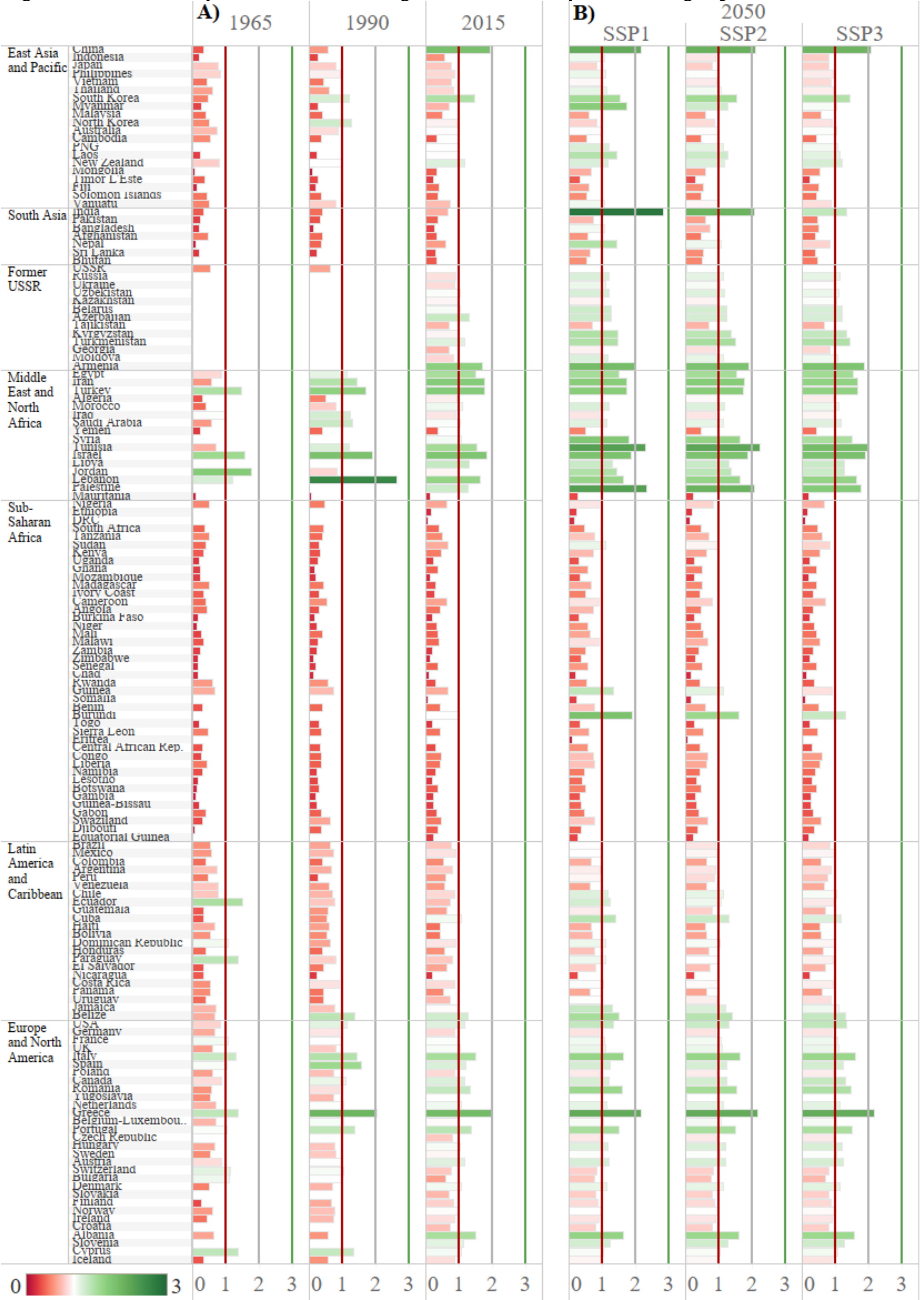

Notes: Countries sorted in descending order by 2010 population. Food availability excludes consumer food waste. 1965 and 1990 values from FAOSTAT Commodity Balance Sheets[27], and 2015 and 2050 values are projections from IMPACT using 3 scenarios (SSP 1–3) using default diets (based on historical trends), and vegetables follow WHO definitions excluding legumes, and starchy roots and tubers. The vertical red, gray, and green lines represent when availability (excl. waste) equals 1X, 2X, and 3X population weighted average recommended consumption levels respectively

Figure S5 All ccountry ratios of fruit & vegetable availability to WHO minimum recommendation (400 g/person/day)

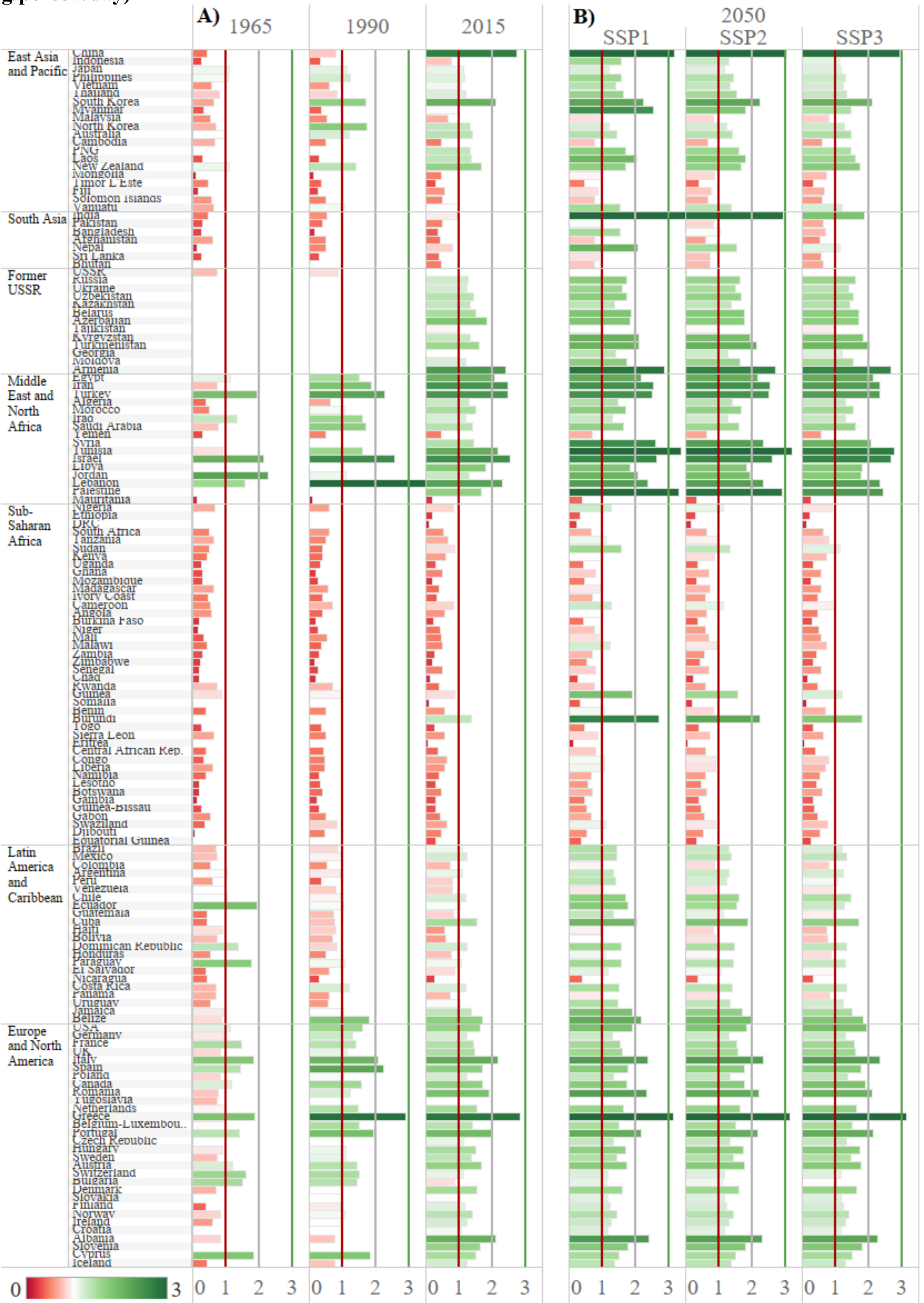

Notes: Countries sorted in descending order by 2010 population. Food availability excludes consumer food waste. 1965 and 1990 values from FAOSTAT Commodity Balance Sheets[27], and 2015 and 2050 values are projections from IMPACT using 3 scenarios (SSP 1–3) using default diets (based on historical trends), and vegetables follow WHO definitions excluding legumes, and starchy roots and tubers. The vertical red, gray, and green lines represent when availability (excl. waste) equals 1X, 2X, and 3X population weighted average recommended consumption levels respectively

Joining NOURISHING Framework and Nuffield Ladder

To assess the current public policy efforts to encourage healthier diets, and specifically encourage consumption of fruits and vegetables, we used the online database of implemented nutrition policies around the world maintained by the World Cancer Research Fund International.[36] This database categorizes the policies using the NOURISHING framework.[37] According to the latest version of the database (updated October 2018), 764 policies relating to healthy eating and reducing obesity have been implemented around the world. We reviewed these policies for specific references of fruit and vegetables, and identified 164 policies that either identified a quantity of fruits and vegetables, or described an activity specifically promoting increased consumption of fruits and vegetables. In this analysis, we excluded policies that described general healthy eating, without specific mention of fruits or vegetables. Several policies were listed in the database under regions (e.g. EU), and in these circumstances we assumed that all countries in the region adopted the identified policy.

The NOURISHING framework categorizes policies in 10 policy areas across three dimensions of the food system (food environment, food system, and consumer behavior), and suggests that changing behavior will require holistic approaches that target change across all dimensions of the food system. Other studies and analyses of policy effectiveness in the healthy diet space have used the Nuffield Ladder[38], which characterizes policies by how interventionist they are in altering the choice environment. These studies suggest that policies on the low end (informational) are less likely to lead to significant behavior change. The two frameworks have a lot in common, and we decided to categorize the policies in the NOURISHING Policy database using both frameworks, to better asses the range across the food system, as well as to the degree of force that these interventions have had.

Mapping the policies from the NOURISHING framework to the Nuffield Ladder was not always straightforward, and depending on the perspective of the actors could be defined in different ways. We decided to categorize the NOURISHING policies based on how they would be perceived by the consumer. For example, the first policy in the NOURISHING framework, "Mandatory nutrient lists on packaged food", could be seen as very prescriptive from the perspective of producers or marketers. However, from the perspective of the consumer this policy is meant to provide information to consumers, and doesn't further alter their choice infrastructure. Thus we mapped this policy to "Provide Information" in the Nuffield Ladder. The mapping we used in this analysis is provided in Table S7

Table S7 Mapping the NOURISHING Framework to the Nuffield Ladder

Domain	Policy Category	Policy Option	Nuffield Ladder
Food Environment	**N**utrition label standards and regulations on the use of claims and implied claims on foods	Mandatory nutrient lists on packaged food	Provide Information
		Trans fats included in mandatory nutrient labels	Provide Information
		Clearly visible "interpretative" labels and warning labels	Provide Information
		On-shelf labelling	Provide Information
		Calorie and nutrient labelling on menus and displays in out-of-home venues	Provide Information
		Warning labels on menus and displays in out-of-home venues	Provide Information
		Rules on nutrient claims	Provide Information
		Rules on health claims	Provide Information
	**O**ffer healthy foods and set standards in public institutions and other specific settings	Fruit and vegetable initiatives in schools	Guide choice by changing default
		Mandatory standards for food available in schools, including restrictions on unhealthy food	Guide choice by changing default
		Mandatory standards for food available in schools and in their immediate vicinity	Guide choice by changing default
		Voluntary guidelines for food available in schools	Guide choice by changing default
		Bans specific vending machines in schools	Eliminate Choice
		Standards in social support programs	Guide choice by changing default
		Standards in other specific locations	Guide choice by changing default
	**U**se economic tools to address food affordability and purchase incentives	Health-related food taxes	Guide choice w/ disincentives
		Voluntary health-related food taxes	Guide choice w/ disincentives
		Increasing import tariffs on specified "unhealthy" food	Guide choice w/ disincentives
		Lowering import tariffs on specified "healthy" food	Guide choice w/ incentives
		Targeted subsidies for healthy food	Guide choice w/ incentives
	**R**estrict food advertising and other forms of commercial promotion	Mandatory regulation of broadcast food advertising to children	Guide choice by changing default
		Mandatory regulation of food advertising on non-broadcast communications channels	Guide choice by changing default
		Mandatory regulation of food advertising through any medium	Guide choice by changing default
		Mandatory regulation of specific marketing techniques	Guide choice by changing default
		Mandatory regulation of marketing of specific food items and beverages	Guide choice by changing default
		Mandatory regulation of food marketing in schools	Guide choice by changing default
		Mandatory requirement that advertisements must carry a health message or warning	Enable Choice
		Voluntary regulation of food advertising on non-broadcast communications channels	Guide choice by changing default
		Government engage with industry to develop self-regulation to restrict food marketing to children	Guide choice by changing default
		Government support voluntary pledges developed by industry	Guide choice by changing default
	**I**mprove the quality of the food supply	Voluntary reformulation of food products	Restrict Choice
		Voluntary commitments to reduce portion sizes	Restrict Choice
		Mandatory limits on level of salt in food products	Restrict Choice
		Mandatory removal of trans fats in food products	Restrict Choice

		Limits on the availability of high-fat meat products	Restrict Choice
		Limits on the availability of high-sugar food products and beverages	Restrict Choice
	**S**et incentives and rules to create a healthy retail environment	Incentives and rules for stores to locate in under-served neighborhoods	Enable Choice
		Initiatives to increase the availability of healthier food in stores and food service outlets	Enable Choice
		Incentives and rules to reduce trans fats in food service outlets	Restrict Choice
		Incentives and rules to offer healthy food options as default in food service outlets	Guide choice by changing default
		Incentives and rules to restrict sugar-sweetened beverage consumption	Restrict Choice
		Incentives and rules to reduce salt in food service outlets	Restrict Choice
		Planning restrictions on food outlets	Restrict Choice
Food Supply	**H**arness supply chain and actions across sectors to ensure coherence with health	Working with food suppliers to provide healthier ingredients	Enable Choice
		Nutrition standards for public procurement	Guide choice by changing default
		Public procurement through "short" chains	Guide choice by changing default
		Supply chain incentives for food production	Enable Choice
		Supporting urban agriculture in health and planning policies	Enable Choice
		Community food production	Enable Choice
		Governance structures for multi-sectoral/stakeholder engagement	Enable Choice
Behavior Change	**I**nform people about food and nutrition through public awareness	Development and communication of food-based dietary guidelines	Provide Information
		Development and communication of guidelines for specific food groups	Provide Information
		Public awareness, mass media and informational campaigns and social marketing on healthy eating	Provide Information
		Public awareness campaigns specific to fruits and vegetables	Provide Information
		Public awareness campaigns concerning specific unhealthy food and beverages	Provide Information
		Public awareness campaigns concerning salt	Provide Information
	**N**utrition advice and counselling in health care settings	Guidelines and programs to provide support in primary care to people who are overweight and obese	Enable Choice
		Nutrition counselling in primary care	Enable Choice
		Training for health professionals	Enable Choice
	**G**ive nutrition education and skills	Nutrition education on curricula	Enable Choice
		Community-based nutrition education	Enable Choice
		Cooking skills	Enable Choice
		Initiatives to train school children on growing food	Enable Choice
		Workplace or community health schemes	Enable Choice
		Training for caterers and food service providers	Enable Choice

References

1 Slavin JL, Lloyd B. Health benefits of fruits and vegetables. *Adv Nutr* 2012; **3**: 506–16.

2 Harper AE. Evolution of Recommended Dietary Allowances—New Directions? *Annu Rev Nutr* 1987; **7**: 509–37.

3 Fischer CG, Garnett T. Plates, pyramids, planet: Developments in national healthy and sustainable dietary guidelines: a state of play assessment. Rome, Italy: FAO, 2016 http://www.fao.org/3/a-i5640e.pdf.

4 CAN. Guide alimentaire du Bénin. 2015 http://poledfn.org (accessed Jan 4, 2019).

5 Hwalla N, Nasreddine L, Farhat Jarrar S. Lebanon Food-Based Dietary Guidelines. Beirut, 2013 http://website.aub.edu.lb/fafs/nfsc/Documents/FBDG English Version.pdf (accessed Jan 4, 2019).

6 Vorster H, Badham J, Venter C. An introduction to the revised food-based dietary guidelines for South Africa. *South African J Clin Nutr* 2013; **26**: S1–164.

7 National Health and Medical Research Council. Australian Dietary Guidelines Summary. Canberra, 2013 www.nhmrc.gov.au/guidelines-publications/n55a (accessed Jan 4, 2019).

8 Wang S-S, Lay S, Yu H-N, Shen S-R. Dietary Guidelines for Chinese Residents (2016): comments and comparisons. *J Zhejiang Univ B* 2016; **17**: 649–56.

9 National Institute of Nutrition. Dietary Guidelines for Indians - A Manual. Hyderabad, 2011 http://ninindia.org/DietaryGuidelinesforNINwebsite.pdf (accessed Jan 4, 2019).

10 Working group on Food-Based Dietary Guidelines for Thai People. Nutrition Flag - Healthy Eating for Thais. Bangkok, 2001 http://www.fao.org/3/a-as979e.pdf (accessed Jan 4, 2019).

11 Health Canada. Eating Well with Canada Food Guide. Ottawa, 2007 www.healthcanada.gc.ca/foodguide (accessed Jan 4, 2019).

12 Calañas A, de Cos AI, López-Nomdedeu C, Ortega R, Palacios N, Vázquez C. Come sano y muévete: decisiones saludables. Madrid, 2008 http://www.aecosan.msssi.gob.es/AECOSAN/docs/documentos/nutricion/educanaos/come_sano_muevete.pdf (accessed Jan 4, 2019).

13 USHHS, USDA. 2015-2020 Dietary Guidelines for Americans. Washington, DC, 2015 http://health.gov/dietaryguidelines/2015/guidelines/. (accessed Jan 4, 2019).

14 Ministerio de Salud de la Nación. Guías alimentarias para la población Argentina. Buenos Aires, 2016 http://www.msal.gob.ar/images/stories/bes/graficos/0000000817cnt-2016-04_Guia_Alimentaria_completa_web.pdf (accessed Jan 4, 2019).

15 Bolivia Ministerio de Salud. Bases técnicas de las guías alimentarias para la población boliviana. La Paz, 2013 http://www.sns.gob.bo (accessed Jan 4, 2019).

16 Academia Nacional de Medicina Mexico. Guías alimentarias y de actividad física. Mexico, DF, 2015 http://guiasalimentacionyactividadfisica.org.mx/wp-content/uploads/2015/10/Guias-alimentarias-y-de-actividad-fisica.pdf (accessed Jan 4, 2019).

17 WHO. Healthy diet. 2018. https://www.who.int/news-room/fact-sheets/detail/healthy-diet (accessed Jan 7, 2019).

18 Lock K, Pomerleau J, Causer L, Mckee M. Low fruit and vegetable consumption. In: Ezzati M, Lopez AD, Rodgers A, Murray CJL, eds. Comparative Quantification of Health Risks Global and Regional Burden of Disease Attributable to Selected Major Risk Factors. Geneva: WHO, 2004: 597–728.

19 Willett W, Rockström J, Loken B, *et al.* Food in the Anthropocene: the EAT–Lancet Commission on healthy diets from sustainable food systems. *Lancet* 2019; **6736**: 3–49.

20 Hasegawa T, Fujimori S, Havlík P, *et al.* Risk of increased food insecurity under stringent global climate change mitigation policy. *Nat Clim Chang* 2018; **8**: 699–703.

21 Stehfest E, van Zeist W-J, Valin H, *et al.* Key determinants of global land-use futures. *Nat Commun*.

22 Delgado C, Rosegrant M, Steinfeld H, Ehui S, Courbois C. Livestock to 2020: The Next Food Revolution. Washington D.C., 1999

http://books.google.com/books?hl=en&lr=&id=MqTT1hsfcy0C&oi=fnd&pg=PR7&dq=Livestock+to+2020+The+Next+Food+Revolution&ots=kxxyaTh11r&sig=tGP7XomQezUhutvZZhqBH24s6b4.

23 Robinson S, Mason-D'Croz D, Islam S, *et al.* The International Model for Policy Analysis of Agricultural Commodities and Trade (IMPACT): Model Description for Version 3. Washington D.C., 2015 DOI:10.2139/ssrn.2741234.

24 Nelson GC, Bogard J, Lividini K, *et al.* Income Growth and Climate Change Effects on Global Nutrition Security to Mid-Century. *Nat Sustain* 2018; **1**. DOI:10.1038/s41893-018-0192-z.

25 Mason-D'Croz D, Robinson S, Dunston S, Sulser TB. A Bayesian methodology for building consistent datasets for structural modeling. Applying information theory to disparate and sparse agricultural datasets for IMPACT. In: 21st Annual Conference on Global Economic Analysis. Cartagena, Colombia: GTAP, 2018: 24.

26 Springmann M, Mason-D'Croz D, Robinson S, *et al.* Global and regional health effects of future food production under climate change: a modelling study. *Lancet* 2016; **387**: 1937–46.

27 Springmann M, Wiebe K, Mason-D'Croz D, Sulser TB, Rayner M, Scarborough P. Health and nutritional aspects of sustainable diet strategies and their association with environmental impacts: a global modelling analysis with country-level detail. *Lancet Planet Heal* 2018; **2**: e451–61.

28 Gustavsson J, Cederberg C, Sonesson U, Emanuelsson A. The methodology of the FAO study: 'Global Food Losses and Food Waste - extent, causes and prevention'. The Swedish Institute for Food and Biotechnology (SIK), 2013 DOI:10.1029/2006JD008332.

29 O'Neill BC, Kriegler E, Ebi KL, *et al.* The roads ahead: Narratives for shared socioeconomic pathways describing world futures in the 21st century. *Glob Environ Chang* 2017; **42**: 169–80.

30 Riahi K, van Vuuren DP, Kriegler E, *et al.* The Shared Socioeconomic Pathways and their energy, land use, and greenhouse gas emissions implications: An overview. *Glob Environ Chang* 2017; **42**: 153–68.

31 KC S, Lutz W. The human core of the shared socioeconomic pathways: Population scenarios by age, sex and level of education for all countries to 2100. *Glob Environ Chang* 2017; **42**: 181–92.

32 IIASA. SSP Public Database. 2018. https://tntcat.iiasa.ac.at/SspV2Preview.

33 Rosegrant MW, Sulser TB, Mason-D'Croz D, *et al.* Quantitative foresight modeling to inform the CGIAR research portfolio. 2017 http://ebrary.ifpri.org/cdm/singleitem/collection/p15738coll2/id/131144.

34 Mason-D'Croz D, Sulser TB, Wiebe K, *et al.* Agricultural investments and hunger in Africa modeling potential contributions to SDG2 – Zero Hunger. *World Dev* 2019; **116**: 38–53.

35 FAO. FAOSTAT Commodity Balances. 2018. http://www.fao.org/faostat/en/#data/BC (accessed Sept 4, 2018).

36 World Cancer Research Fund International. NOURISHING Policy Database. 2018. https://www.wcrf.org/int/policy/nourishing-database (accessed Dec 1, 2018).

37 Hawkes C, Jewell J, Allen K. A food policy package for healthy diets and the prevention of obesity and diet-related non-communicable diseases: the NOURISHING framework. *Obes Rev* 2013; **14**: 159–68.

38 Nuffield Council on Bioethics. Policy process and pracice. In: Public health: ethical issues. London, 2007: 29–47.
